# Neurophysiological Characterization of ADHD in Children Using EEG Signals: A Machine Learning Approach to Executive Function Networks

**DOI:** 10.3390/s26175684

**Published:** 2026-09-07

**Authors:** Diana Beatriz Gutiérrez-Jácome, Rosalynn Argelia Campos-Ortuño, José Eduardo Pardo-Valenzuela, Óscar Wladimir Gómez-Morales

**Affiliations:** 1 Carrera de Gestión del Desarrollo Infantil Familiar Comunitario, Facultad de Ciencias de la Educación e Idiomas, Universidad Estatal Península de Santa Elena, La Libertad 240250, Ecuador; 2 Universidad Santo Tomas—Seccional Tunja, Boyacá 150001, Colombia; docpedagogia.neurociencia@ustatunja.edu.co; 3 Universidad de Salamanca, 37008 Salamanca, Spain; rosecampos@usal.es; 4 Facultad de Sistemas y Telecomunicaciones, Universidad Estatal Península de Santa Elena, La Libertad 240250, Ecuador; oscargomez@upse.edu.ec

**Keywords:** attention deficit hyperactivity disorder (ADHD), electroencephalography (EEG), machine learning, EEG classification, subject-wise validation, individual alpha frequency (IAF), spectral features, executive functions, neurophysiology

## Abstract

Attention Deficit Hyperactivity Disorder (ADHD) is a prevalent neurodevelopmental disorder whose clinical assessment relies mainly on behavioral and neuropsychological evaluation. This study evaluates a subject-wise machine learning framework for distinguishing children with ADHD from healthy controls using multichannel EEG-derived features. The public dataset comprised 121 participants (61 ADHD and 60 controls), with 19-channel EEG recordings sampled at 128 Hz. Signals were segmented into 4-s windows with 50% overlap, and statistical and spectral features were extracted, including mean, standard deviation, and theta-, alpha-, and beta-band power. Support Vector Machine (SVM), Random Forest (RF), Gradient Boosting (GB), and Logistic Regression (LR) were evaluated using strict subject-wise separation. RF achieved the highest Accuracy (0.8099), F1-score (0.8160), Balanced Accuracy (0.8097), and MCC (0.6204), whereas SVM obtained the highest Sensitivity (0.8525) and ROC-AUC (0.8527). An additional subject-specific analysis based on individual alpha frequency (IAF) was performed to account for inter-individual spectral variability; mean IAF values were 8.8320 Hz for ADHD and 8.8833 Hz for controls, and the individualized-band analysis did not improve classification performance. Bootstrap confidence intervals and non-parametric tests indicated comparable performance among RF, SVM, and GB. Frontal and fronto-central channels, particularly Fz, showed the greatest model-derived contribution. Overall, the framework provides a reproducible subject-wise EEG classification approach, although external validation on independent cohorts remains necessary before clinical application.

## 1. Introduction

Attention Deficit Hyperactivity Disorder (ADHD) is one of the most prevalent neurodevelopmental disorders during childhood and adolescence, affecting approximately 5–7% of the pediatric population worldwide [1,2,3]. Its persistence across development can substantially affect cognitive functioning, academic performance, social adaptation, and quality of life [4,5].

The clinical expression of ADHD is markedly heterogeneous and includes different symptom presentations, developmental trajectories, and frequent neurodevelopmental or psychiatric comorbidities. This variability complicates differential diagnosis and limits the extent to which a single behavioral or neurophysiological marker can represent the disorder. Consequently, current research increasingly focuses on identifying quantitative biomarkers that can complement, rather than replace, conventional clinical assessment.

From a neuropsychological perspective, ADHD is associated with impairments in executive functions, particularly sustained attention, working memory, inhibitory control, planning, cognitive flexibility, and behavioral self-regulation [6]. These difficulties have been related to inefficient functioning of distributed neural systems involved in goal-directed behavior and cognitive control.

Despite advances in the understanding of its neurobiological basis, ADHD diagnosis continues to rely primarily on clinical interviews, behavioral rating scales, and diagnostic criteria established by international guidelines such as the DSM-5 and ICD-11 [7,8,9]. Although these instruments provide essential clinical information, their application depends partly on clinician judgment and on reports from parents, teachers, or caregivers, which may introduce variability into the diagnostic process.

In addition, methodological studies based on historical public datasets must consider that some cohorts were originally characterized using earlier diagnostic standards, such as DSM-IV. Differences between historical and current diagnostic criteria may affect the definition of clinical presentations and should therefore be acknowledged when interpreting computational findings obtained from previously collected data.

Electroencephalography (EEG) has emerged as a useful non-invasive technique for investigating neurophysiological alterations associated with ADHD [7,10]. Compared with other functional neuroimaging modalities, EEG provides high temporal resolution, relatively low acquisition costs, portability, and practical applicability in pediatric populations [11,12]. These characteristics enable the study of cortical oscillatory activity associated with attention, working memory, inhibitory control, and other executive processes.

Studies comparing children with ADHD and healthy controls have reported differences in spectral power, neural synchronization, functional connectivity, and spatiotemporal patterns of EEG activity [13,14,15,16,17]. Theta, alpha, and beta oscillations have received particular attention because of their relationships with attentional regulation, cortical activation, working memory, and response control [18,19].

However, conventional fixed frequency boundaries may not fully represent the developmental and inter-individual variability of pediatric EEG. Cortical oscillatory frequencies evolve throughout childhood, and children with ADHD may exhibit a lower dominant alpha frequency than typically developing peers. Accordingly, a fixed boundary between theta and alpha activity may classify physiologically different oscillations within the same predefined band and partially contribute to heterogeneous findings across studies [20,21,22]. These observations do not invalidate conventional band-power analysis, but they emphasize that its results should be interpreted cautiously and within the context of developmental brain maturation.

The interpretation of pediatric EEG is also affected by physiological artifacts. Low-amplitude ocular activity and frontal or temporal electromyographic contamination may overlap with theta and beta frequencies and cannot always be eliminated by conventional amplitude thresholds or frequency filtering alone. Such activity may influence spectral estimates, particularly during cognitive tasks or in participants with elevated motor activity or psychoemotional stress. Therefore, artifact-related limitations must be explicitly considered when interpreting scalp-level EEG biomarkers [23,24].

Recent evidence also indicates that ADHD-related alterations are not limited to isolated changes in spectral power but involve distributed interactions among frontal, frontocentral, and parietal regions associated with executive control [25,26]. Nevertheless, scalp EEG channels represent electrical potentials recorded at the sensor level and cannot, by themselves, establish the precise cortical or subcortical generators of the observed activity. Formal source-localization analyses would be required to attribute sensor-level patterns to specific anatomical structures or large-scale brain networks.

In parallel, advances in artificial intelligence and machine learning have expanded the analytical possibilities of multichannel EEG by enabling the identification of complex temporal and spectral patterns that may be difficult to detect using conventional statistical approaches [27]. Supervised algorithms such as Support Vector Machine (SVM), Random Forest (RF), Gradient Boosting (GB), and Logistic Regression (LR) have been applied to ADHD classification using features derived from EEG recordings [28,29,30,31].

However, the reported performance of these approaches is difficult to compare directly because studies differ in acquisition conditions, channel configurations, participant characteristics, preprocessing methods, feature definitions, and validation procedures. Furthermore, the limited availability of large, harmonized, multicenter pediatric EEG datasets restricts external validation and remains a major obstacle to establishing the generalizability of computational models across institutions, age groups, recording systems, and clinical populations.

A recurrent methodological limitation concerns the partitioning of segmented EEG recordings. Continuous signals are frequently divided into multiple overlapping windows that are subsequently distributed randomly between training and testing sets. Although this strategy increases the apparent number of observations, it can introduce information leakage when correlated segments from the same participant are included in both partitions [32,33]. Under these conditions, models may learn participant-specific patterns instead of disorder-related characteristics, producing overly optimistic performance estimates.

To reduce this risk, the present study uses a strict subject-wise evaluation scheme in which all segments belonging to a given participant remain exclusively within either the training or testing partition during each validation iteration. In this manuscript, the term “subject-wise validation” refers to evaluation on participants not included in the corresponding training partition. This design assesses generalization to unseen individuals within the available dataset, although it does not constitute external validation on an independent cohort.

Another important limitation in the current literature is the emphasis placed on predictive metrics without equivalent attention to the physiological interpretation of model outputs. Accuracy or ROC-AUC values alone do not establish which EEG features, scalp regions, or cognitive mechanisms contribute to classification decisions [34,35]. This limitation is particularly relevant in clinical research, where computational outputs should be interpretable and considered alongside behavioral, neuropsychological, and medical information.

Accordingly, the present study addresses two complementary research needs. First, it evaluates statistical and spectral EEG descriptors under strict subject-wise separation to obtain a more conservative estimate of classification performance. Second, it examines the relative contribution of EEG channels and the organization of the extracted feature space to provide a sensor-level interpretation of the patterns associated with ADHD. The purpose is not to propose a standalone diagnostic system, but to investigate whether an interpretable and reproducible analytical framework can provide complementary neurophysiological information relevant to executive dysfunction.

The central research question is whether a compact set of interpretable EEG descriptors can retain meaningful discriminative information when evaluated under a leakage-controlled subject-wise protocol. On this basis, the study examines the following hypotheses: (H1) statistical and spectral EEG descriptors contain measurable information that differentiates ADHD and control participants under subject-wise evaluation; (H2) the comparative behavior of nonlinear and linear classifiers reflects differences in their capacity to model the multivariate structure of the selected EEG features; (H3) frontal and frontocentral sensors provide a comparatively greater contribution to classification, without implying direct localization of the underlying neural sources; and (H4) combining predictive evaluation, confidence intervals, statistical comparisons, and sensor-level interpretation provides a more informative assessment than reporting classification accuracy alone.

The primary objective of this study is to develop and evaluate a machine learning framework for the neurophysiological characterization and classification of children with ADHD using multichannel EEG signals. The implemented pipeline includes signal preprocessing, temporal segmentation, statistical and spectral feature extraction, supervised classification, subject-wise evaluation, statistical analysis, and sensor-level interpretation of the resulting patterns.

The remainder of this paper is organized as follows. Section 2 describes the methodology for EEG preprocessing, feature extraction, classification, and statistical analysis. Section 3 presents the experimental setup and the characteristics of the public EEG dataset. Section 4 reports the predictive, statistical, and sensor-level results. Section 5 discusses the findings in relation to previous studies, methodological limitations, developmental factors, and potential confounders. Finally, Section 6 summarizes the principal conclusions and outlines directions for external and multicenter validation.

## 2. Materials and Methods

### 2.1. EEG Data Acquisition

The EEG data acquisition stage corresponds to the initial step of the proposed methodological framework. In this phase, multichannel electroencephalographic recordings were obtained using a standard configuration of the international 10–20 system, considering a total of 19 EEG channels.

The channel configuration and sampling frequency were defined by the original acquisition protocol of the publicly available dataset and were not selected or modified by the authors of the present study. The recordings were sampled at 128 Hz, providing a Nyquist frequency of 64 Hz. This sampling rate is sufficient for the analysis of the theta, alpha, and beta frequency bands considered in this work; however, it limits the reliable examination of higher-frequency activity, such as gamma oscillations, and the detailed temporal microstructure that may be captured using acquisition systems operating at 250 Hz, 500 Hz, or higher sampling rates.

The 19-channel international 10–20 montage provides broad sensor-level coverage of frontal, central, temporal, parietal, and occipital scalp regions and is consistent with routine clinical EEG configurations. Nevertheless, its spatial resolution is lower than that of high-density EEG systems, and the recorded scalp potentials cannot be directly attributed to specific cortical or subcortical generators without a formal source-localization procedure.

Accordingly, the present analysis is restricted to sensor-level neurophysiological patterns within the frequency range supported by the original recording protocol.

This stage provides the raw EEG input signal, represented as continuous multichannel recordings, which is subsequently subjected to filtering, normalization, segmentation, and feature extraction processes to feed the machine learning models.

### 2.2. EEG Signal Preprocessing

#### 2.2.1. Band-Pass Filtering

Band-pass filtering retains only the relevant frequency components of the EEG signal by removing both low frequencies (baseline drift and slow physiological artifacts) and high frequencies (muscle or electromagnetic noise). In this work, a range of 1–45 Hz was used, which covers the main neurophysiological bands (delta, theta, alpha, and beta).

From a mathematical perspective, filtering can be modeled as a convolution between the original signal x(t) and the impulse response of the filter h(t):

(1) $$y\left(t\right)=x\left(t\right)*h\left(t\right)=\int_{-\infty }^{\infty }x\left(\tau \right)\;h\left(t-\tau \right)\;d\tau$$

In the frequency domain, the ideal band-pass filter is expressed as:

(2) $$H\left(f\right)=\left\{\begin{array}{cc} 1, & f_{L}\leq \left|f\right|\leq f_{H}\\ 0, & \mathrm{otherwise} \end{array}\right.$$

where $f_{L}$ and $f_{H}$ correspond to the lower and upper cutoff frequencies, respectively [36].

#### 2.2.2. Band-Stop (Notch) Filter

The notch filter is used to remove narrowband frequency interference, mainly power line noise (50 Hz or 60 Hz depending on the country). This type of filter strongly attenuates a central frequency $f_{0}$ while preserving the rest of the spectrum.

The transfer function of a typical notch filter can be expressed as:

(3) $$H\left(z\right)=\frac{1-2\cos\left(\omega_{0}\right)z^{-1}+z^{-2}}{1-2r\cos\left(\omega_{0}\right)z^{-1}+r^{2}z^{-2}}$$

where:

- $\omega_{0}=\frac{2\pi f_{0}}{f_{s}}$ is the normalized frequency.
- *r* controls the bandwidth of the filter.
- $f_{s}$ is the sampling frequency.

This design allows selective removal of interference without significantly affecting the relevant EEG bands [37].

#### 2.2.3. Z-Score Normalization

Z-score normalization is applied to standardize the EEG signal channel by channel, removing scale differences between subjects and sessions, which is crucial for machine learning models.

The transformation is defined as:

(4) $$z=\frac{x-\mu }{\sigma }$$

where:

- *x* is the original signal value.
- μ is the channel mean.
- σ is the standard deviation.

This operation produces a signal with zero mean and unit variance:

(5) $$\mu_{z}=0,\;\sigma_{z}=1$$

which improves numerical stability and the convergence of classification algorithms [38].

The adopted preprocessing pipeline was designed to preserve the original characteristics of the publicly available EEG dataset while reducing common sources of signal contamination through band-pass filtering, notch filtering, and channel-wise normalization. No Independent Component Analysis (ICA) or other advanced artifact removal techniques were applied, as these procedures were not part of the original preprocessing protocol adopted in this study. Consequently, residual ocular and electromyographic (EMG) artifacts may still be present in some recordings, particularly in frontal electrodes where these physiological interferences are more pronounced. Although these artifacts cannot be completely eliminated using conventional frequency filtering, the application of a strict subject-wise validation strategy ensures that all EEG segments from the same participant remain within the same data partition during model evaluation, thereby preventing information leakage between the training and testing sets. Therefore, although subject-wise validation prevents information leakage between training and testing sets, the potential contribution of residual physiological artifacts to the classification results cannot be completely excluded. This limitation is considered during the interpretation of the neurophysiological findings and is further discussed in Section 5.

#### 2.2.4. Feature Extraction

The objective of the feature extraction stage was to transform each normalized EEG window into a compact numerical representation capable of preserving the most relevant temporal and spectral information associated with ADHD. Feature extraction was performed independently for each segmented EEG window after channel-wise normalization, reducing amplitude-scale differences between channels and subjects.

Feature extraction was performed independently for each segmented EEG window after the preprocessing stage. No additional window-wise Z-score normalization was applied at this stage. Statistical and spectral descriptors were computed from each EEG window and subsequently concatenated into feature vectors. Before classifier training, the resulting feature vectors were standardized using the mean and standard deviation estimated exclusively from the training data within each cross-validation fold, and the same transformation was then applied to the corresponding test data. This procedure prevented information leakage between the training and testing partitions.

Subsequently, five complementary features were extracted from each EEG channel, including two statistical descriptors and three spectral descriptors. Considering the 19 EEG channels available in the dataset, the final feature vector contained a total of 95 features for every EEG window.

The statistical descriptors were computed as follows.

The mean value was calculated as

(6) $$\mu =\frac{1}{N}\sum_{i=1}^{N}x_{i}$$

where *N* denotes the number of samples within the EEG window.

The standard deviation was computed as

(7) $$\sigma =\sqrt{\frac{1}{N}\sum_{i=1}^{N}{\left(x_{i}-\mu \right)}^{2}}$$

These statistical descriptors characterize the central tendency and signal variability, providing information about amplitude fluctuations associated with cortical activity.

The spectral descriptors were estimated using Welch’s Power Spectral Density (PSD) method, which provides a robust estimate of the frequency content while reducing the variance of the spectral estimate through segment averaging [39].

The PSD is expressed as

(8) $$P_{xx}\left(f\right)=\frac{1}{LU}\sum_{l=0}^{L-1}{\left|\sum_{n=0}^{M-1}x_{l}\left(n\right)w\left(n\right)e^{-j2\pi fn}\right|}^{2}$$

where *L* represents the number of overlapping segments, *M* is the segment length, w(n) is the analysis window, and *U* is the normalization factor.

The spectral power within each frequency band was then obtained by integrating the estimated PSD over the corresponding frequency interval:

(9) $$BP=\int_{f_{1}}^{f_{2}}P_{xx}\left(f\right)\;df$$

where $\left(f_{1},f_{2}\right)$ defines the limits of the analyzed EEG band.

In this work, three conventional EEG frequency bands were considered:

- Theta band: 4–8 Hz.
- Alpha band: 8–13 Hz.
- Beta band: 13–30 Hz.

These frequency bands were selected because they have been consistently associated with attention regulation, executive function, working memory, and cognitive control in children with ADHD [40,41,42].

Finally, the feature vector corresponding to each EEG window was constructed by concatenating the five descriptors extracted from every channel according to

(10) $$F={\left[\mu ,\sigma ,\theta ,\alpha ,\beta \right]}_{1}⊕{\left[\mu ,\sigma ,\theta ,\alpha ,\beta \right]}_{2}⊕\cdots ⊕{\left[\mu ,\sigma ,\theta ,\alpha ,\beta \right]}_{19}$$

where ⊕ denotes vector concatenation. Consequently, each EEG window was represented by a 95-dimensional feature vector (19 channels × 5 features), which was subsequently used as input for the supervised machine learning classifiers.

#### 2.2.5. Channel Importance Estimation

To facilitate the neurophysiological interpretation of the extracted EEG features, channel importance was estimated using the feature importance scores provided by the Random Forest classifier. The analysis was performed within the same Stratified Group K-Fold cross-validation framework adopted for model evaluation, ensuring that feature importance was estimated exclusively from the training subjects in each fold and avoiding information leakage.

For each fold, a Random Forest classifier composed of 300 decision trees was trained using the extracted feature vectors. The importance associated with each feature was computed according to the mean decrease in impurity (MDI), which measures the contribution of a feature to the reduction of node impurity throughout the ensemble.

The importance of the *j*-th feature is defined as

(11) $$FI_{j}=\frac{1}{T}\sum_{t=1}^{T}\sum_{n\in t}p\left(n\right)\;\Delta I\left(n\right),$$

where *T* denotes the total number of trees, p(n) is the proportion of samples reaching node *n*, and ΔI(n) represents the impurity reduction produced by splitting with feature *j*.

The feature importance vectors obtained from the five cross-validation folds were subsequently averaged to obtain a stable estimate of the contribution of each extracted descriptor:

(12) $${\bar{FI}}_{j}=\frac{1}{K}\sum_{k=1}^{K}FI_{j}^{\left(k\right)},$$

where K=5 corresponds to the number of folds.

Since each EEG channel was represented by five descriptors (mean, standard deviation, theta power, alpha power, and beta power), the channel importance was computed as the average importance of its associated features:

(13) $$CI_{c}=\frac{1}{5}\sum_{m=1}^{5}{\bar{FI}}_{c,m},$$

where $CI_{c}$ denotes the importance assigned to channel *c*.

Finally, the channel importance values were normalized using Min-Max normalization,

(14) $$CI_{c}^{norm}=\frac{CI_{c}-\min\left(CI\right)}{\max(CI)-\min(CI)},$$

allowing all channels to be represented within the interval [0,1]. These normalized values were subsequently projected onto the standard international 10–20 scalp montage to generate a topographic representation, while the frontal-channel ranking was obtained directly from the non-normalized channel importance values.

### 2.3. Machine Learning Models

#### 2.3.1. Support Vector Machine with Radial Basis Function (SVM-RBF)

The Support Vector Machine (SVM) model aims to find an optimal hyperplane that maximizes the separation between classes in a feature space. When the data are not linearly separable, a kernel function is employed to project the samples into a higher-dimensional space. In this work, the Gaussian Radial Basis Function (RBF) kernel was used due to its ability to model complex nonlinear relationships present in EEG signals.

The decision function of the SVM is defined as:

(15) $$f\left(x\right)=\mathrm{sign}\left(\sum_{i=1}^{N}\alpha_{i}y_{i}K\left(x_{i},x\right)+b\right)$$

where:

- $\alpha_{i}$ are the Lagrange multipliers.
- $y_{i}$ corresponds to the class labels.
- $x_{i}$ are the support vectors.
- *b* is the bias term of the model.
- $K(x_{i},x)$ is the kernel function.

The RBF kernel is mathematically expressed as:

(16) $$K\left(x_{i},x_{j}\right)=\exp\left(-\gamma ∥x_{i}-x_{j}{∥}^{2}\right)$$

where γ controls the influence of each sample in the decision space [43].

#### 2.3.2. Random Forest

Random Forest is an ensemble learning algorithm based on the combination of multiple randomly constructed decision trees. Each tree is trained using random subsets of data samples and features, reducing overfitting and improving the generalization capability of the model [44].

The final prediction of the model is obtained through majority voting:

(17) $$\hat{y}=\mathrm{mode}\left\{h_{1}\left(x\right),h_{2}\left(x\right),\ldots ,h_{T}\left(x\right)\right\}$$

where:

- $h_{t}\left(x\right)$ represents the prediction of the *t*-th tree.
- *T* is the total number of trees.

Node splitting is commonly performed using the Gini index:

(18) $$G=1-\sum_{i=1}^{C}p_{i}^{2}$$

where:

- $p_{i}$ represents the probability of belonging to class *i*.
- *C* is the total number of classes.

#### 2.3.3. Gradient Boosting

Gradient Boosting is a sequential ensemble learning method that iteratively constructs weak models by progressively minimizing a loss function through gradient descent optimization. Each new tree attempts to correct the errors made by the previous models.

The iterative update of the model is expressed as:

(19) $$F_{m}\left(x\right)=F_{m-1}\left(x\right)+\eta h_{m}\left(x\right)$$

where:

- $F_{m}\left(x\right)$ is the model at iteration *m*.
- $h_{m}\left(x\right)$ is the weak learner trained on the residual errors.
- η is the learning rate.

The optimization process is based on minimizing a loss function L(y,F(x)):

(20) $$F^{*}=\arg\min_{F}\sum_{i=1}^{N}L\left(y_{i},F\left(x_{i}\right)\right)$$

This approach allows the model to capture complex and nonlinear relationships present in EEG data [45].

#### 2.3.4. Logistic Regression

Logistic Regression is a probabilistic model commonly used for binary classification tasks. It estimates the probability of belonging to a particular class using a sigmoid function applied to a linear combination of features.

The predicted probability is defined as:

(21) $$P\left(y=1|x\right)=\frac{1}{1+e^{-(w^{T}x+b)}}$$

where:

- *w* represents the weight vector.
- *x* corresponds to the input feature vector.
- *b* is the bias term of the model.

The loss function used during training is the binary cross-entropy loss:

(22) $$L=-\frac{1}{N}\sum_{i=1}^{N}\left[y_{i}\log\left({\hat{y}}_{i}\right)+\left(1-y_{i}\right)\log\left(1-{\hat{y}}_{i}\right)\right]$$

This model is widely used as a baseline approach in EEG classification due to its simplicity and interpretability.

#### 2.3.5. Validation Strategy

To ensure a reliable assessment of model generalization and to prevent information leakage between training and testing data, two complementary subject-level validation strategies were adopted according to the objective of each experiment.

The primary evaluation of the proposed machine learning models was performed using Stratified Group K-Fold cross-validation with five folds. In this strategy, each participant was considered as an independent group, ensuring that all EEG windows belonging to the same subject remained exclusively in either the training or testing partition during each fold. Consequently, no EEG segments from the same participant were simultaneously present in both subsets, preventing information leakage and providing a realistic estimation of the model generalization capability.

Formally, let $S=\{s_{1},s_{2},\ldots ,s_{N}\}$ denote the set of subjects. The validation strategy partitions the subjects into K=5 mutually exclusive folds:

(23) $$S=\overset{K}{\underset{k=1}{⋃}}S_{k},\;S_{i}\cap S_{j}=⌀,\;i\neq j,$$

where all EEG windows extracted from a subject $s_{i}$ belong exclusively to the same fold. For each iteration, one fold was used for testing while the remaining folds were employed for training:

(24) $$D_{train}=\underset{i\neq k}{⋃}S_{i},\;D_{test}=S_{k}.$$

This protocol ensures strict subject-wise separation between the training and testing partitions during model evaluation and provides class-balanced partitions while preserving the original class distribution [43].

Since feature extraction was performed using overlapping temporal windows, subject-wise partitioning was essential to avoid artificially inflated performance caused by highly correlated segments originating from the same EEG recording. Thus, all reported performance metrics correspond to previously unseen subjects rather than randomly selected EEG segments.

As a complementary validation procedure, Leave-One-Subject-Out (LOSO) cross-validation was applied exclusively to the Support Vector Machine classifier for generating the subject-level confusion matrix presented in Section 4. Under this protocol, one subject was iteratively selected for testing while the remaining participants were used for training:

(25) $$D_{test}=\left\{s_{i}\right\},\;D_{train}=S\setminus \left\{s_{i}\right\},$$

ensuring that every participant was evaluated exactly once. Since each subject generated multiple EEG windows, the final subject label was obtained by majority voting over the window-level predictions:

(26) $${\hat{y}}_{subject}=\mathrm{mode}\left({\hat{y}}_{1},{\hat{y}}_{2},\ldots ,{\hat{y}}_{M}\right),$$

where *M* denotes the number of windows extracted from the corresponding subject.

The two validation strategies were used for complementary purposes. Stratified Group K-Fold cross-validation with strict subject-wise separation was adopted as the primary validation strategy for the comparative evaluation of the four machine learning models and for the Bootstrap confidence intervals and statistical comparisons. LOSO validation was used as a complementary subject-level evaluation for the confusion matrix and for the computational cost analysis, in which each participant was iteratively held out for testing. Both strategies maintained complete subject independence between training and testing data. However, both procedures represent internal validation within the available public dataset and do not constitute external validation. Therefore, evaluation on independent and multicenter cohorts remains necessary to assess the generalizability of the proposed framework.

#### 2.3.6. Statistical Analysis

To evaluate the robustness of the classification models and determine whether the observed differences in their performance were statistically significant, complementary statistical analyses were conducted on the evaluation metrics obtained during the cross-validation process. Specifically, the Bootstrap method was employed to estimate 95% confidence intervals for the principal performance metrics, while the non-parametric Friedman test followed by the Wilcoxon signed-rank post-hoc test was used to compare the performance of the evaluated classifiers.

#### 2.3.7. Bootstrap Confidence Interval Estimation

The stability of the classification metrics was assessed using the Bootstrap method with 5000 resampling iterations performed with replacement on the predictions obtained during cross-validation. For each bootstrap sample, the evaluation metrics were recalculated, generating an empirical distribution for each estimator. Subsequently, the 95% confidence interval was obtained from the 2.5th and 97.5th percentiles of the bootstrap distribution.

Let

(27) $$X=\{x_{1},x_{2},\ldots ,x_{n}\}$$

be the original dataset, and let

(28) $$\hat{\theta }=f\left(X\right)$$

denote the estimator of a classification metric (e.g., Accuracy, F1-score, or ROC-AUC).

In the Bootstrap procedure, *B* random samples with replacement are generated.

(29) $$X^{*(1)},X^{*(2)},\ldots ,X^{*(B)},$$

where, in this study,

(30) B=5000.

Each bootstrap sample produces a new estimate:

(31) $${\hat{\theta }}^{*(b)}=f\left(X^{*(b)}\right),\;b=1,\ldots ,B.$$

Finally, the 95% confidence interval is estimated using the percentile method.

(32) $$CI_{95\%}=\left[Q_{2.5}\left({\hat{\theta }}^{*}\right),Q_{97.5}\left({\hat{\theta }}^{*}\right)\right],$$

where $Q_{2.5}$ and $Q_{97.5}$ denote the 2.5th and 97.5th percentiles of the bootstrap distribution, respectively.

This procedure provides a robust estimate of the variability of the classification metrics without assuming any specific probability distribution and is widely recommended for biomedical classification problems involving moderate sample sizes [46].

#### 2.3.8. Friedman Test

To determine whether statistically significant overall differences existed among the four evaluated classifiers, the non-parametric Friedman test was applied. This test is appropriate for comparing multiple algorithms evaluated on the same validation folds.

Let:

- *N* be the number of validation blocks (folds).
- *k* be the number of classifiers.
- $R_{j}$ be the sum of the ranks assigned to classifier *j*.

The Friedman test statistic is defined as

(33) $$\chi_{F}^{2}=\frac{12N}{k(k+1)}\sum_{j=1}^{k}R_{j}^{2}-3N\left(k+1\right).$$

The null hypothesis assumes that all classifiers exhibit equivalent performance.

(34) $$H_{0}:M_{1}=M_{2}=\cdots =M_{k},$$

whereas the alternative hypothesis states that at least one classifier performs significantly differently from the others.

A significance level of

(35) α=0.05

was adopted throughout the statistical analysis [47,48].

#### 2.3.9. Post-Hoc Analysis Using the Wilcoxon Signed-Rank Test

Whenever the Friedman test revealed statistically significant differences (p<0.05), pairwise comparisons between classifiers were performed using the Wilcoxon signed-rank test.

Let

(36) $$d_{i}=x_{i}-y_{i}$$

represent the difference between the performance metrics obtained by two classifiers on the same validation block.

After removing zero differences and ranking the absolute values $|d_{i}|$, the Wilcoxon statistic is computed as

(37) $$W=\min(W^{+},W^{-}),$$

where

(38) $$W^{+}=\sum_{d_{i}>0}r_{i},$$

and

(39) $$W^{-}=\sum_{d_{i}<0}r_{i}.$$

The null hypothesis assumes that the median of the paired differences is equal to zero.

(40) $$H_{0}:\tilde{d}=0,$$

indicating that both classifiers exhibit statistically equivalent performance.

Pairwise comparisons were performed for all possible combinations of the evaluated classifiers (Support Vector Machine, Random Forest, Gradient Boosting, and Logistic Regression), and the corresponding *p*-values were reported to identify statistically significant differences between models [49].

### 2.4. Evaluation Metrics

To quantitatively evaluate the performance of the classification models, several metrics derived from the confusion matrix were computed. Let TP, TN, FP, and FN denote the numbers of true positives, true negatives, false positives, and false negatives, respectively.

#### 2.4.1. Accuracy

Accuracy measures the proportion of correctly classified samples among all evaluated samples [50].

(41) $$\mathrm{Accuracy}=\frac{TP+TN}{TP+TN+FP+FN}$$

#### 2.4.2. Precision

Precision quantifies the proportion of correctly identified ADHD subjects among all samples predicted as ADHD [51].

(42) $$\mathrm{Precision}=\frac{TP}{TP+FP}$$

#### 2.4.3. Sensitivity (Recall)

Sensitivity evaluates the ability of the classifier to correctly identify ADHD subjects [51].

(43) $$\mathrm{Sensitivity}=\frac{TP}{TP+FN}$$

#### 2.4.4. Specificity

Specificity measures the capability of the model to correctly recognize healthy control subjects [50].

(44) $$\mathrm{Specificity}=\frac{TN}{TN+FP}$$

#### 2.4.5. F1-Score

The F1-score corresponds to the harmonic mean of Precision and Recall [50].

(45) $$F1\text{-}\mathrm{score}=2\times \frac{\mathrm{Precision}\times \mathrm{Recall}}{\mathrm{Precision}+\mathrm{Recall}}$$

An equivalent formulation is given by:

(46) $$F1\text{-}\mathrm{score}=\frac{2TP}{2TP+FP+FN}$$

#### 2.4.6. Balanced Accuracy

Balanced Accuracy is particularly useful when class distributions are not perfectly balanced and is defined as [50,51]:

(47) $$\mathrm{Balanced}\;\mathrm{Accuracy}=\frac{\mathrm{Sensitivity}+\mathrm{Specificity}}{2}$$

or equivalently,

(48) $$\mathrm{Balanced}\;\mathrm{Accuracy}=\frac{1}{2}\left(\frac{TP}{TP+FN}+\frac{TN}{TN+FP}\right)$$

#### 2.4.7. Matthews Correlation Coefficient (MCC)

The Matthews Correlation Coefficient provides a balanced evaluation even when class sizes differ substantially [51,52].

(49) $$\mathrm{MCC}=\frac{TP\times TN-FP\times FN}{\sqrt{\left(TP+FP)(TP+FN)(TN+FP)(TN+FN\right)}}$$

The MCC ranges from:

(50) −1≤MCC≤1

where:

- MCC=1 indicates perfect classification.
- MCC=0 indicates random prediction.
- MCC=−1 indicates complete disagreement.

#### 2.4.8. Receiver Operating Characteristic (ROC)

The ROC curve is generated by plotting the True Positive Rate (TPR) against the False Positive Rate (FPR) [52].

(51) $$\mathrm{TPR}=\frac{TP}{TP+FN}$$

(52) $$\mathrm{FPR}=\frac{FP}{FP+TN}$$

#### 2.4.9. Area Under the Curve (AUC)

The Area Under the ROC Curve (AUC) measures the overall discriminative ability of the classifier across all possible decision thresholds.

(53) $$\mathrm{AUC}=\int_{0}^{1}\mathrm{TPR}\left(\mathrm{FPR}\right)\;d\left(\mathrm{FPR}\right)$$

where larger AUC values indicate better discrimination between ADHD and Control subjects [51,52].

## 3. Experimental Set-Up

**Figure 1:**
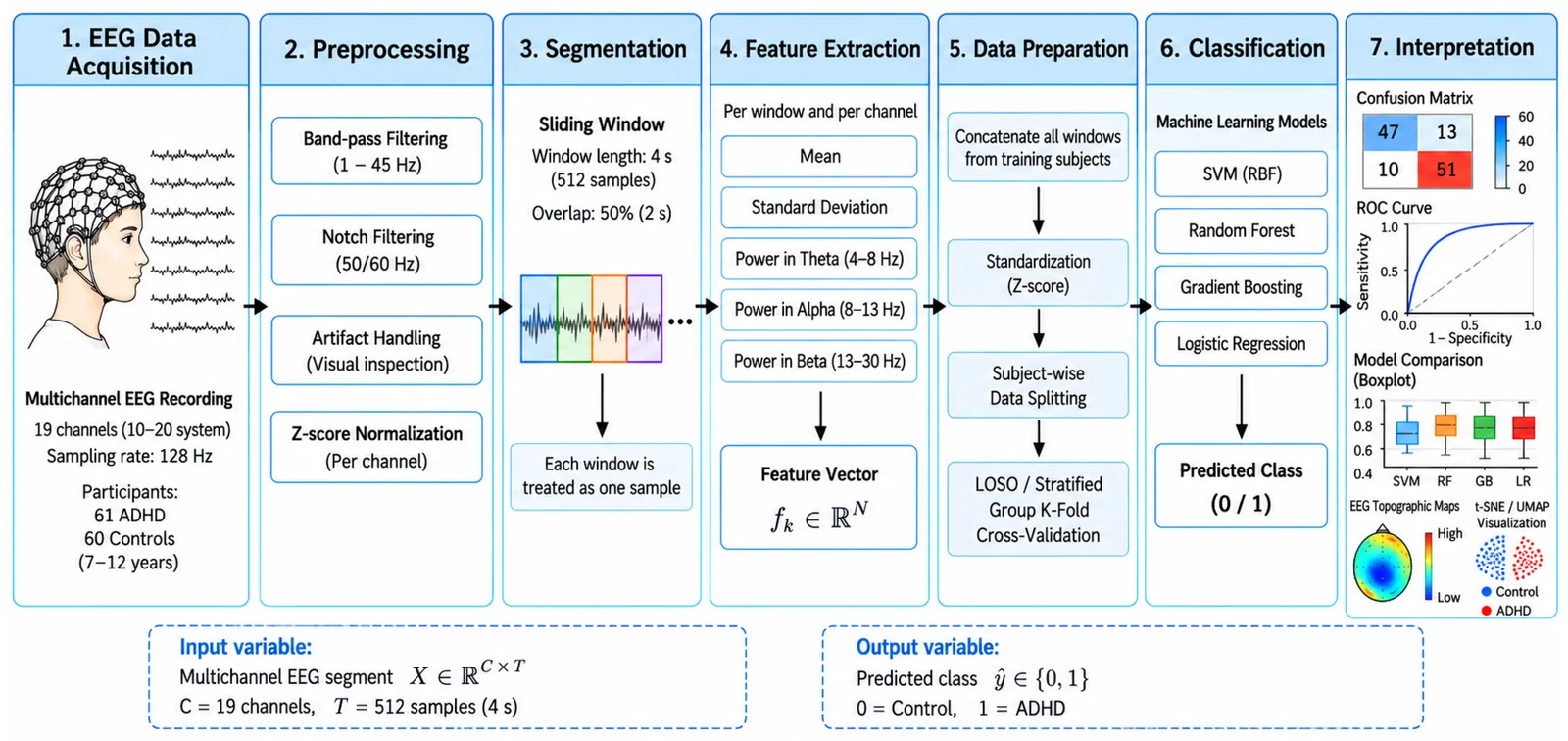
General workflow of the proposed study. Channel-wise Z-score normalization is applied during EEG preprocessing. After feature extraction, the resulting feature vectors are standardized using the mean and standard deviation estimated exclusively from the training data within each cross-validation fold, and the same transformation is subsequently applied to the corresponding test data. All data splitting is performed subject-wise using Stratified Group K-Fold cross-validation.

The experiments conducted in this study were designed to evaluate the capability of different machine learning models to classify subjects with ADHD and control subjects using electroencephalographic signals. Figure 1 illustrates the overall workflow of the study. The experimental framework was developed considering a complete EEG signal processing pipeline, including acquisition, preprocessing, segmentation, feature extraction, and supervised classification. The EEG signals were acquired using a multichannel system based on the international 10–20 standard, employing 19 channels and a sampling frequency of 128 Hz. Subsequently, the signals were subjected to a preprocessing stage composed of band-pass filtering between 1–45 Hz, notch filtering for power-line noise removal, and channel-wise Z-score normalization. In order to increase the number of available samples and capture relevant temporal information, the EEG signals were segmented using sliding windows of 4 s (512 samples) with 50% overlap. Each EEG window was treated as an individual input instance for feature extraction and classification. However, all windows derived from the same participant were kept within the same subject-wise partition, preventing segments from the same individual from being simultaneously included in the training and testing sets. From each EEG window, statistical and spectral features related to neurophysiological brain activity were extracted. The considered features included the mean, standard deviation, and spectral power within theta (4–8 Hz), alpha (8–13 Hz), and beta (13–30 Hz) frequency bands, due to their relevance in studies associated with executive functions and cognitive alterations related to ADHD.

To account for inter-individual variability in EEG spectral characteristics, an additional subject-specific analysis was performed using the individual alpha frequency (IAF). For each participant, the power spectral density (PSD) was estimated using Welch’s method across the 19 EEG channels, and the channel-wise spectra were averaged to obtain a representative subject-level spectrum. The IAF was identified as the dominant spectral peak within the 7–14 Hz interval.

(54) $${\mathrm{IAF}}_{s}=\underset{f\in [7,14]\;\mathrm{Hz}}{\mathrm{arg max}}\;{\bar{P}}_{s}\left(f\right)$$

where ${\bar{P}}_{s}\left(f\right)$ represents the power spectral density averaged across the 19 EEG channels for subject *s*, estimated using Welch’s method.

Individualized frequency bands were subsequently defined relative to each participant’s IAF as follows: theta, IAF −6 to IAF −2 Hz; alpha, IAF −2 to IAF +2 Hz; and beta, IAF +2 to IAF +20 Hz. Spectral power was then recalculated within these individualized frequency ranges for each EEG window and channel. The remaining feature extraction and subject-wise classification procedures were maintained unchanged, allowing direct comparison with the conventional fixed-band analysis. Subsequently, the extracted features were concatenated and standardized before training the classification models. To ensure a robust and unbiased evaluation, a Stratified Group K-Fold cross-validation strategy with strict subject-wise separation was adopted. This validation scheme guarantees that all EEG segments belonging to the same participant are assigned exclusively to either the training or the testing set, thereby preventing information leakage and providing a more realistic estimate of the model generalization capability. The present study was conducted using a publicly available EEG dataset acquired and released by the original investigators. Therefore, the EEG acquisition protocol, participant recruitment, diagnostic procedures, and recording conditions were established prior to this work and were not modified by the authors. Consequently, the objective of the present research was not to generate a new clinical dataset but to develop, implement, and evaluate a reproducible machine learning framework for the neurophysiological characterization and classification of ADHD using standardized EEG recordings. All preprocessing, feature extraction, classification, statistical analyses, and interpretation presented in this study were performed exclusively on the publicly available dataset.

Finally, four supervised models were evaluated: Support Vector Machine with RBF kernel (SVM-RBF), Random Forest, Gradient Boosting, and Logistic Regression. The performance of the classifiers was analyzed using classification metrics, including confusion matrices, ROC curves, and statistical comparisons between models, allowing the identification of the most effective strategies for EEG-based neurophysiological characterization of ADHD.

### 3.1. EEG Dataset for ADHD

The dataset used in this study corresponds to the public *EEG Dataset for ADHD*, available at the: Kaggle ADHD EEG Dataset, https://www.kaggle.com/datasets/danizo/eeg-dataset-for-adhd?utm_source=chatgpt.com (accessed on 1 September 2026) [53]. This dataset is openly accessible and can be used by the scientific community for research in EEG signal analysis and neurological disorder classification.

### 3.2. EEG Dataset Description

The dataset used in this study consists of a set of electroencephalographic signals acquired from a pediatric population with the aim of analyzing neurophysiological alterations associated with ADHD. The dataset is composed of multichannel recordings obtained from both clinically diagnosed ADHD subjects and healthy control subjects without reported neurological history, enabling the development of computational models oriented toward the automatic identification of discriminative brain activity patterns. The ADHD diagnoses were established by a psychiatrist with extensive clinical experience, following the diagnostic criteria defined in the DSM-IV. The diagnosed participants had received pharmacological treatment with Ritalin for a period not exceeding six months. In contrast, the subjects belonging to the control group had no history of neurological or psychiatric disorders. Table 1 summarizes the demographic characteristics of the participants, as well as the parameters used during EEG signal acquisition.

**Figure 2:**
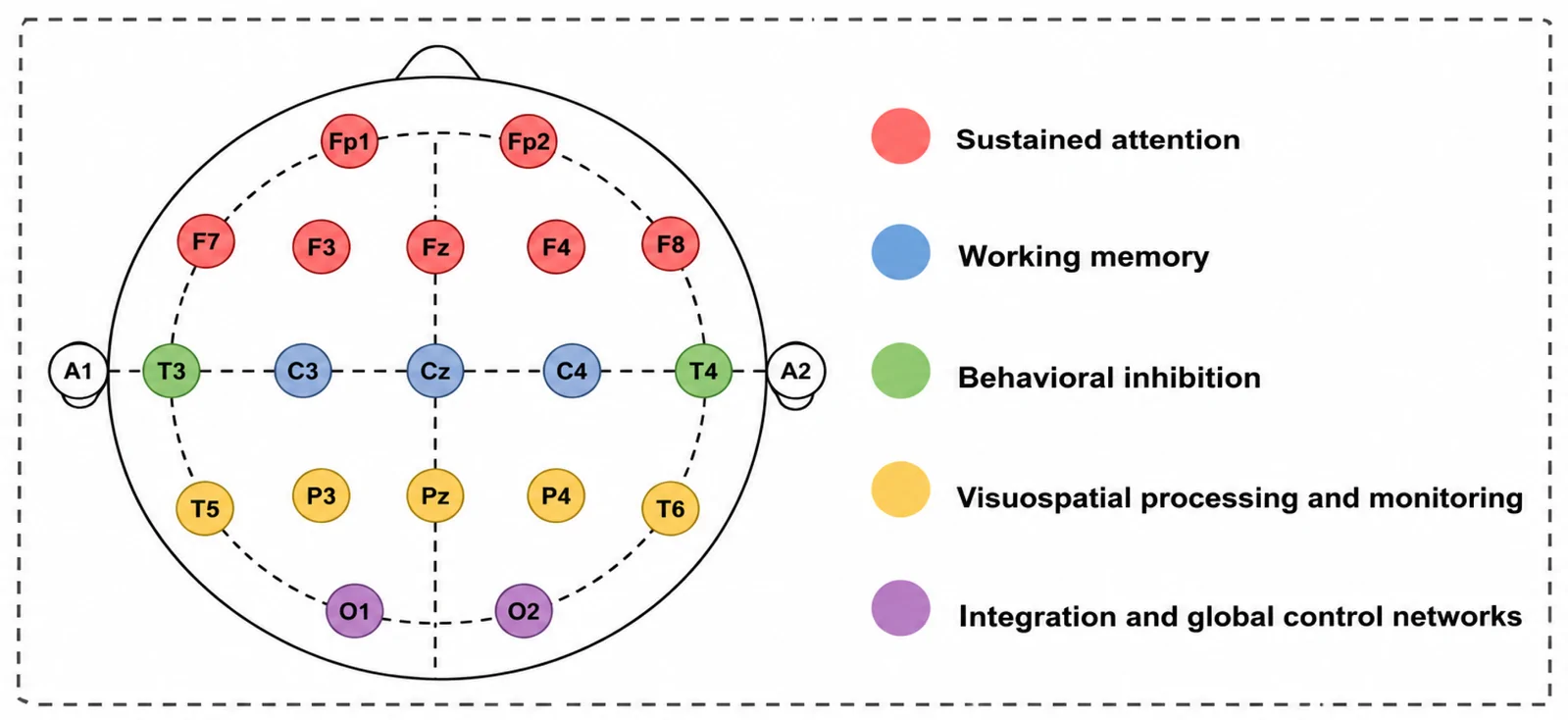
Standard 10–20 system with 19 EEG channels used in the original acquisition protocol. The montage includes frontal, central, temporal, parietal, and occipital electrodes, while A1 and A2 correspond to the reference electrodes located on the earlobes. The colors indicate the functional predominance associated with the represented scalp regions in relation to attention, working memory, behavioral inhibition, visuospatial processing, and executive control.

**Figure 3:**
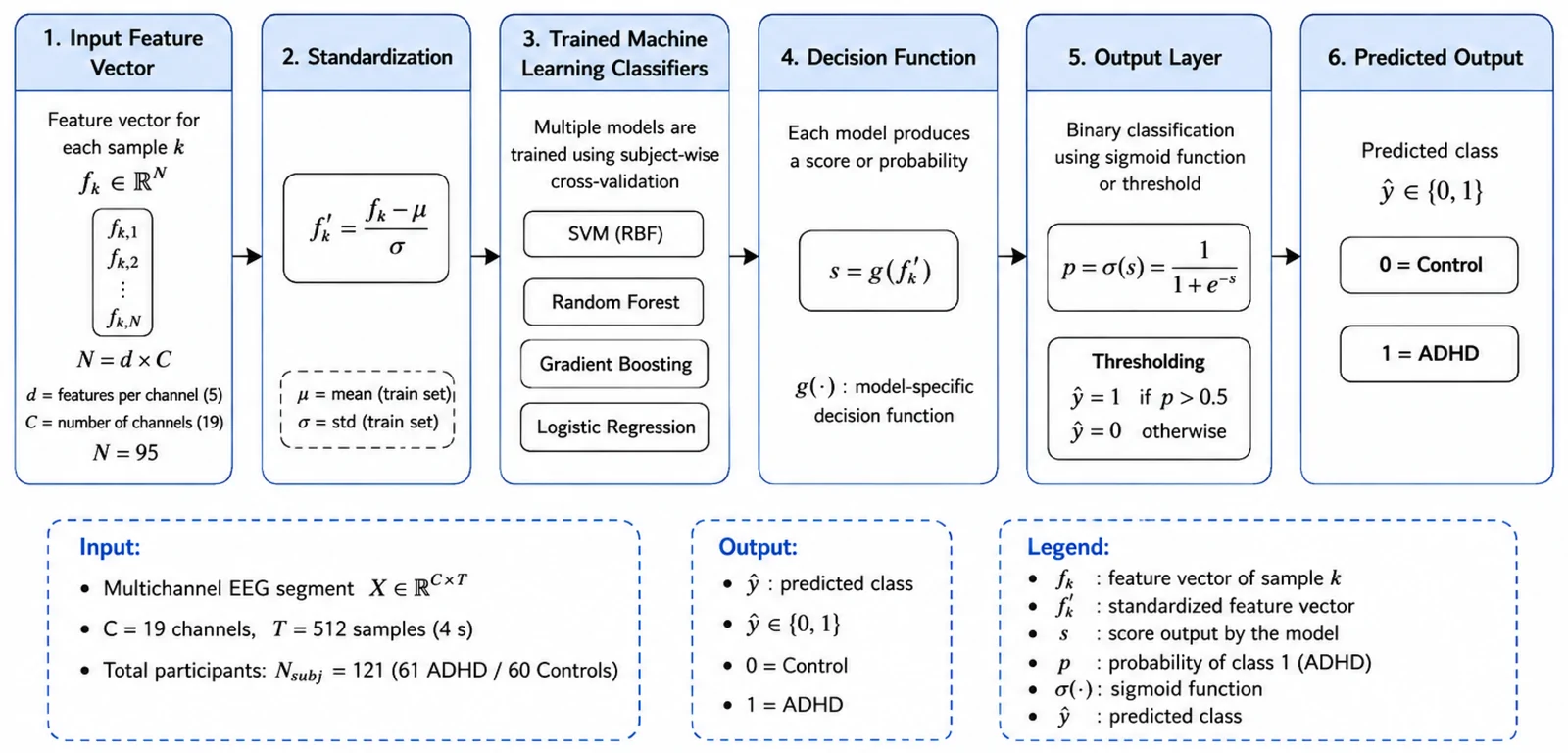
General architecture of the proposed methodological framework for the neurophysiological characterization and classification of ADHD using EEG signals.

The EEG recordings were acquired using a configuration based on the international 10–20 system, as illustrated in Figure 2, employing 19 channels strategically distributed over frontal, central, temporal, parietal, and occipital scalp regions. This spatial distribution enables the capture of neuronal activity associated with executive functions, sustained attention, working memory, and behavioral inhibition, processes that have been extensively linked to functional alterations in patients with ADHD. The general architecture of the proposed methodological framework is summarized in Figure 3.

The database includes EEG signals from children approximately 7 to 12 years of age, an age range in which the neurocognitive symptoms of ADHD exhibit high clinical and behavioral relevance. The EEG recordings were obtained while participants performed a visual attention task in which they were instructed to count cartoon characters presented on a screen. The recording duration varied across participants according to their individual response speed, following the acquisition protocol established in the original dataset.

Each EEG recording contains multivariate temporal series acquired at a sampling frequency of 128 Hz, providing sufficient temporal resolution for the analysis of low- and mid-frequency brain oscillations. This resolution preserves relevant spectral information within the theta, alpha, and beta frequency bands, which are of particular interest in neurophysiological ADHD studies due to their association with attention mechanisms and cognitive regulation processes.

The public EEG dataset employed in this study was originally collected from children clinically diagnosed according to the DSM-IV diagnostic criteria available at the time of data acquisition. The ADHD group included participants who had received methylphenidate (Ritalin) treatment for no longer than six months, whereas the control group consisted of children without a reported history of neurological or psychiatric disorders. Furthermore, the dataset presents a moderate imbalance in the sex distribution between groups, reflecting the original composition of the cohort rather than any selection criterion introduced in this study. Consequently, these characteristics should be considered when interpreting the reported classification performance and the potential generalizability of the proposed framework to other pediatric populations.

### 3.3. EEG Signal Segmentation

**Figure 4:**
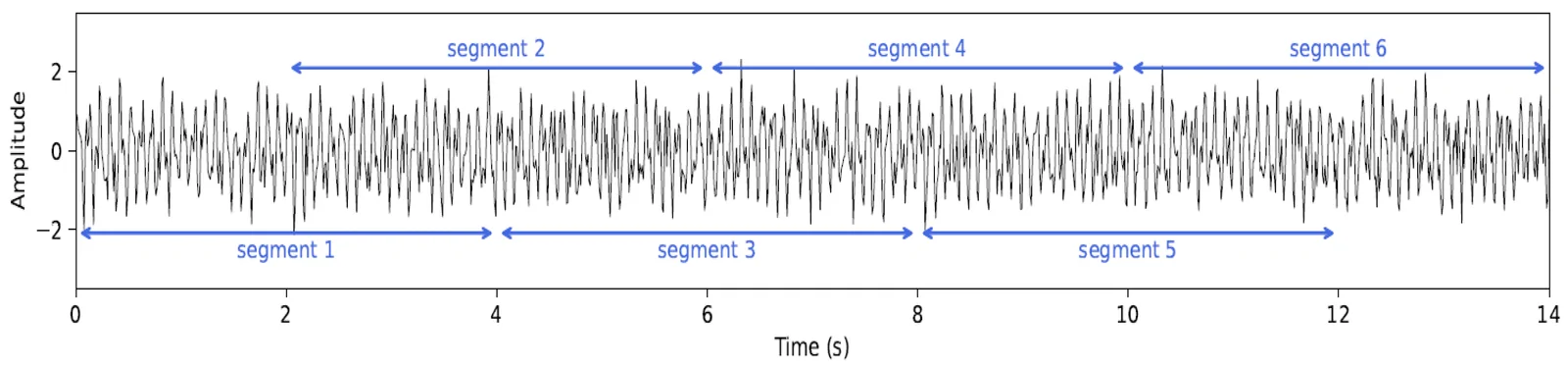
Segmentation of raw EEG signals into 4-s windows (512 samples at 128 Hz) with 50% overlap. Sliding windows advance every 2 s, yielding consecutive partially overlapping epochs that preserve temporal continuity and improve the representation of dynamic neurophysiological patterns. This segmentation strategy increases the number of training samples while maintaining relevant spectral and temporal EEG information associated with executive function alterations in ADHD.

Continuous EEG signals were segmented into temporal epochs to generate suitable samples for the training and evaluation of the classification models. For this purpose, a sliding window strategy with a duration of 4 s was employed, corresponding to 512 samples considering a sampling frequency of 128 Hz. Additionally, a 50% overlap was applied between consecutive windows, which increased the temporal density of the samples and preserved the dynamic continuity of brain activity without losing relevant neurophysiological information. Consequently, each new window started every 2 s, as illustrated in Figure 4, allowing the capture of subtle temporal variations present in EEG signals associated with cognitive processes related to ADHD.

As a result of the segmentation process, a total of 3657 EEG segments were obtained for the control group, with an average of (56±15) segments per subject, whereas the ADHD group generated 4622 segments, with an average of (75±29) segments per subject. Each EEG epoch was treated as an individual input instance during the classification process, increasing the number of window-level observations available for training the supervised models.

To avoid information leakage and ensure a robust evaluation of the classifiers, the identity of each subject was strictly preserved throughout the entire experimental process. In particular, epochs belonging to the same individual were never simultaneously mixed between the training and testing sets during cross-validation. This subject-wise separation strategy ensures that the models learn generalizable neurophysiological patterns rather than subject-specific characteristics, thereby improving the reliability and clinical validity of the obtained results.

Although the adopted experimental protocol ensured strict subject-wise separation throughout training and testing, the proposed framework was evaluated using a single publicly available EEG dataset. Consequently, the reported results should be interpreted within the context of the acquisition protocol, participant characteristics, and recording conditions of this database. While the obtained findings demonstrate the robustness of the proposed methodology under controlled subject-wise validation, further validation using independent multicenter datasets acquired with different EEG systems and clinical populations is necessary to establish the generalizability and clinical applicability of the proposed framework.

## 4. Results

### 4.1. EEG Signal Representation and Visualization

**Figure 5:**
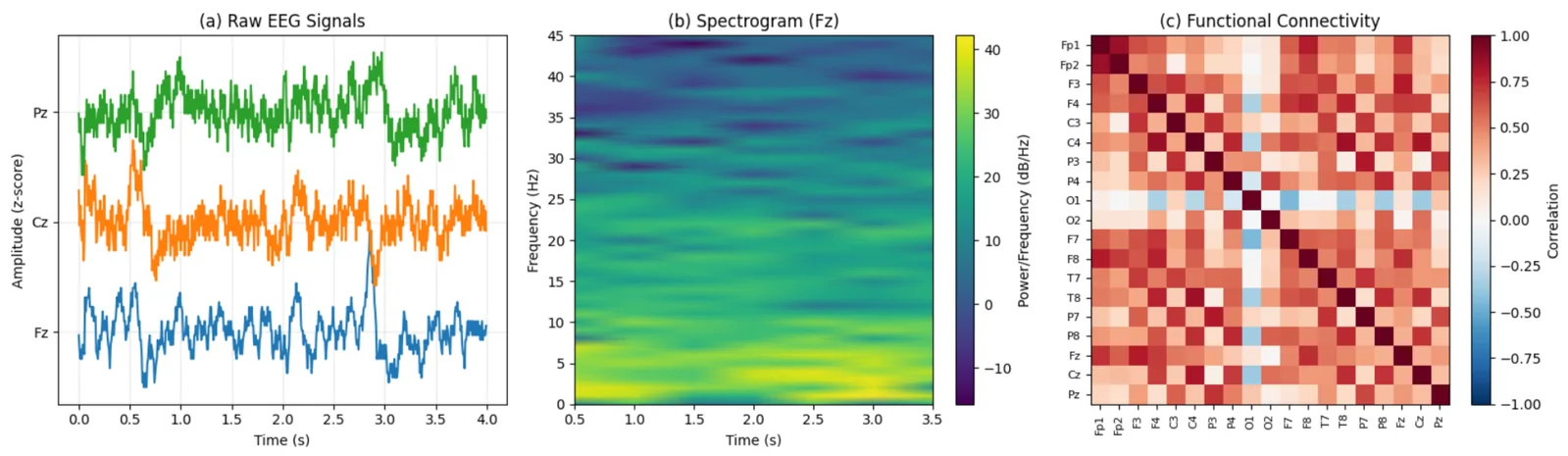
Multi-domain EEG representation of a representative ADHD subject: (**a**) normalized raw EEG signals from Fz, Cz, and Pz channels in the time domain; (**b**) time–frequency spectrogram of the Fz channel showing spectral power distribution; and (**c**) functional connectivity matrix between 19 EEG channels based on Pearson correlation.

Figure 5 presents three complementary representations of EEG signals obtained from a representative subject belonging to the ADHD group, illustrating the temporal, spectral, and functional characteristics of the recorded brain activity. These visualizations are intended to provide an intuitive representation of the EEG signals analyzed in this study and to illustrate the different signal domains considered throughout the proposed methodological framework. They should be interpreted as descriptive examples rather than quantitative evidence supporting the classification results.

Figure 5a shows the temporal representation of the raw EEG signal corresponding to channels Fz, Cz, and Pz, selected due to their functional relevance within the fronto-central and parietal midline regions. These cortical regions are closely associated with sustained attention, cognitive control, and working memory processes. To improve visual comparison between channels, the signals were normalized using Z-score normalization and represented within a 4-s temporal window. The displayed waveforms preserve the temporal dynamics of spontaneous EEG activity while facilitating qualitative comparison of amplitude fluctuations across the selected channels.

Figure 5b presents the time–frequency spectrogram of channel Fz, obtained through spectral analysis over the same segmented EEG window. This representation illustrates the distribution of spectral power as a function of time within the frequency range from 0 to 45 Hz. The spectrogram highlights the temporal evolution of oscillatory activity within the theta, alpha, and beta frequency bands, which constitute the spectral features subsequently extracted for machine learning analysis. It should be noted that this visualization represents a single EEG segment and therefore is not intended to infer population-level neurophysiological differences between ADHD and control subjects.

Figure 5c shows the functional connectivity matrix estimated across the 19 EEG channels using Pearson correlation. This representation allows the degree of linear association to be evaluated between different cortical regions during the analyzed EEG segment. The connectivity matrix provides a qualitative visualization of the spatial organization of inter-channel correlations within the representative EEG recording. Since connectivity measures were not incorporated as input features for the classification models, this representation is included exclusively to illustrate the spatial relationships present in the analyzed EEG signals and should not be interpreted as evidence of altered functional connectivity associated with ADHD.

### 4.2. Confusion Matrix Analysis

**Figure 6:**
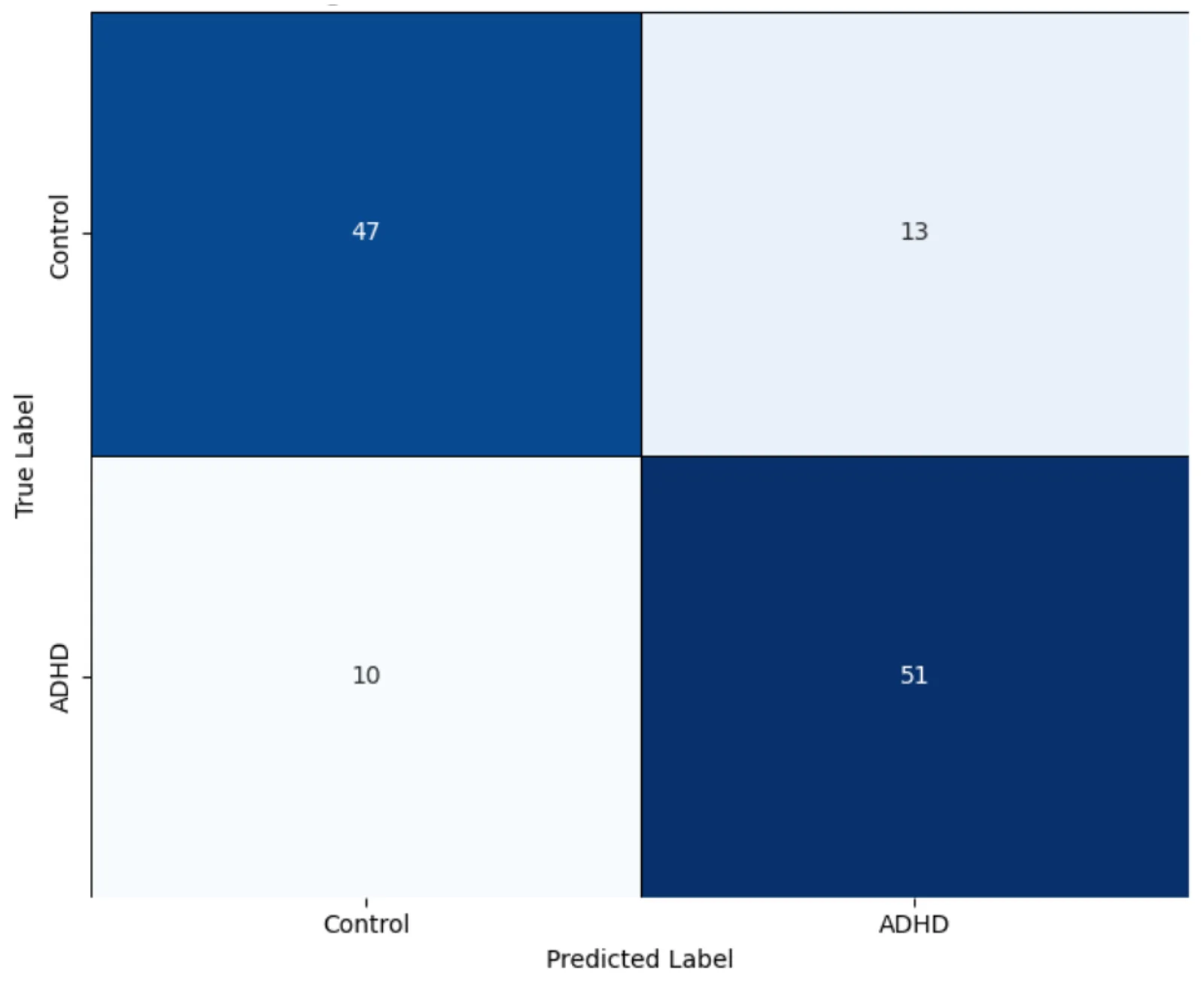
Confusion matrix of the ADHD vs. Control classification model showing the absolute number of correctly and incorrectly classified subjects.

**Figure 7:**
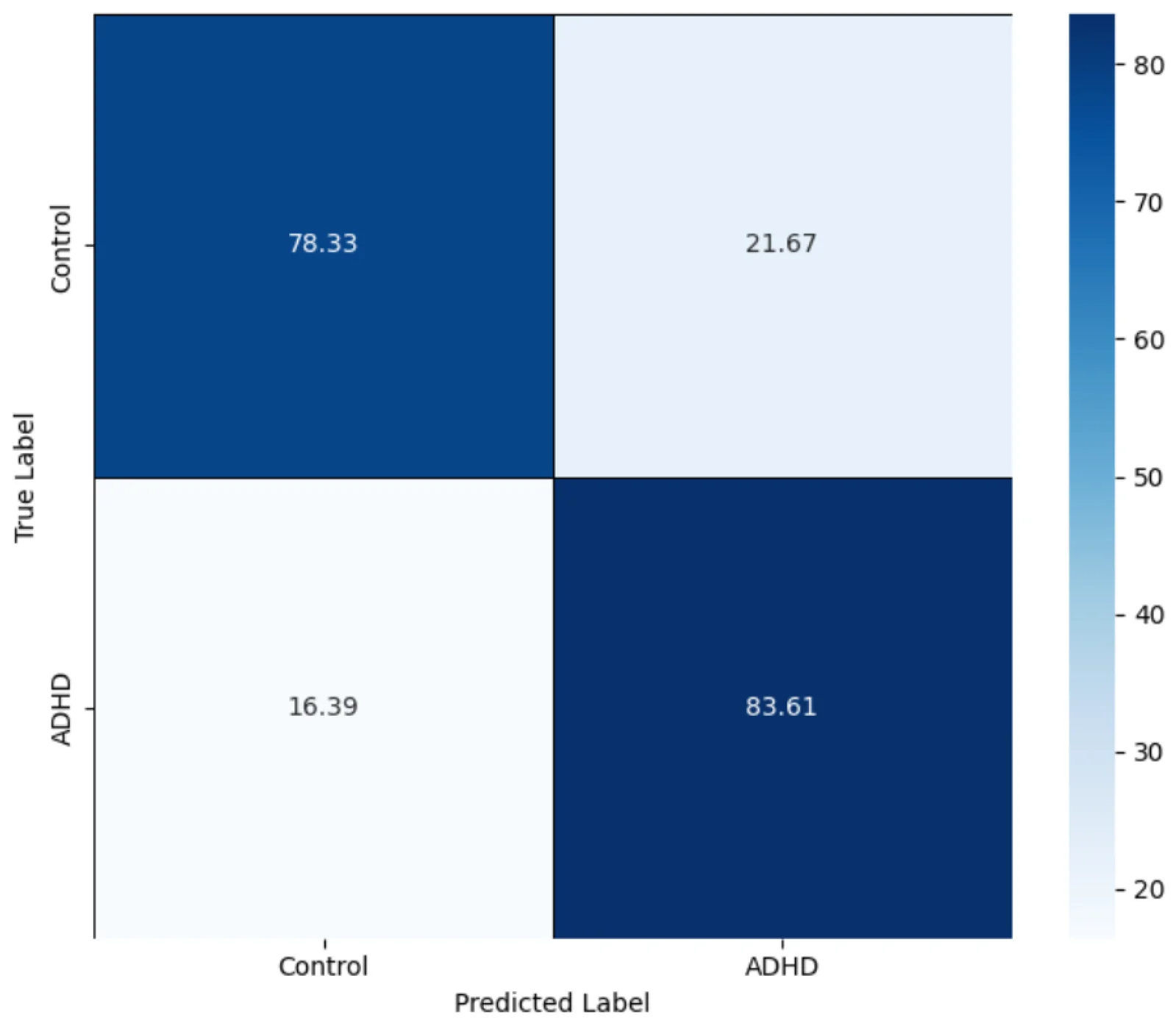
Normalized confusion matrix of the ADHD vs. Control classification model expressed as percentage of classification performance for each class.

Figure 6 and Figure 7 present the confusion matrix obtained for the ADHD versus Control classification model, allowing the predictive performance of the classifier to be evaluated in both absolute and relative terms. These results correspond to the subject-level evaluation performed using the Leave-One-Subject-Out (LOSO) validation strategy described in Section 2, where each participant was iteratively excluded from the training set and classified independently. These representations provide a detailed view of the model behavior for both classes and allow identification of the balance between correct predictions and classification errors.

Figure 6 shows the confusion matrix expressed as the absolute number of correctly and incorrectly classified subjects. It can be observed that the model correctly classified 47 subjects from the control group and 51 subjects from the ADHD group, while 13 control subjects were incorrectly classified as ADHD and 10 ADHD subjects were misclassified as control. Overall, these results indicate that the proposed framework achieved a balanced classification performance for both diagnostic groups, with a slightly higher ability to correctly identify ADHD subjects than healthy controls.

Figure 7 presents the same confusion matrix normalized as percentages relative to the total number of subjects in each class. The results indicate that the model achieved a classification sensitivity of 83.61% for the ADHD group and a specificity of 78.33% for the control group. Additionally, the observed error rates were 16.39% for ADHD subjects misclassified as control and 21.67% for control subjects misclassified as ADHD. The normalized confusion matrix confirms that the classifier maintained a consistent level of performance across both classes, supporting the robustness of the subject-wise evaluation while also highlighting the remaining overlap between ADHD and control subjects, which is expected given the intrinsic variability of pediatric EEG signals.

### 4.3. Receiver Operating Characteristic (ROC) Analysis

**Figure 8:**
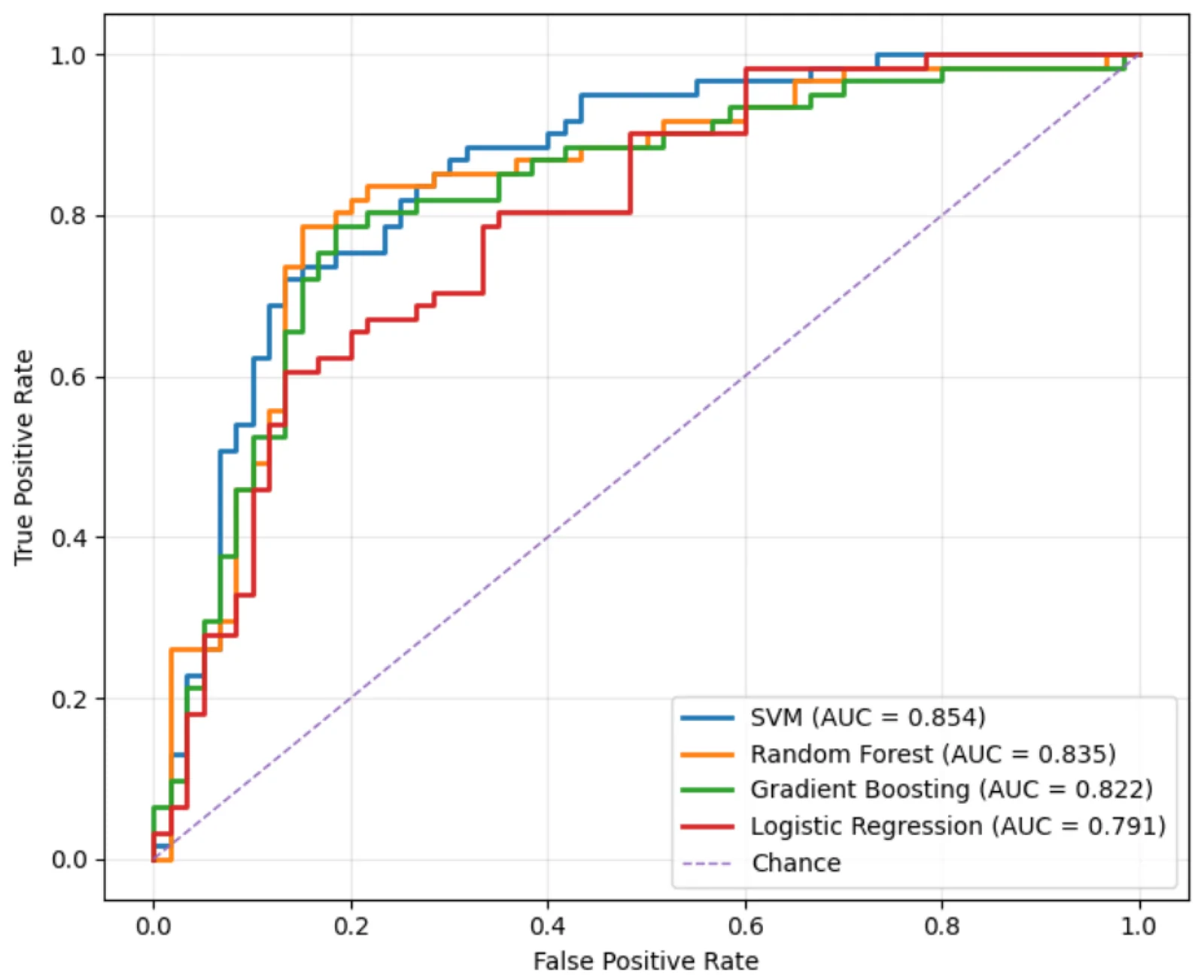
Receiver Operating Characteristic (ROC) curves comparing the classification performance of SVM, Random Forest, Gradient Boosting, and Logistic Regression models for ADHD versus Control discrimination based on EEG features.

Figure 8 presents the Receiver Operating Characteristic (ROC) curves obtained for the four evaluated classification models: Support Vector Machine (SVM), Random Forest (RF), Gradient Boosting (GB), and Logistic Regression (LR). The ROC analysis provides a threshold-independent evaluation of the discriminative capability of each classifier by representing the relationship between the True Positive Rate (TPR) and the False Positive Rate (FPR) over a range of decision thresholds.

The ROC curves indicate that the evaluated classifiers achieved different levels of discrimination between children with ADHD and healthy control subjects. Among the evaluated models, SVM exhibited the highest discriminative performance, whereas Random Forest achieved a closely comparable ROC profile. Gradient Boosting showed intermediate performance, while Logistic Regression consistently produced the lowest discriminative capability across the evaluated thresholds. The numerical AUC values corresponding to each classifier are summarized in Table 2.

It can be observed that all ROC curves remain above the diagonal reference line corresponding to random classification, indicating that each model extracted meaningful information from the EEG feature space. The relatively similar ROC profiles obtained by SVM, Random Forest, and Gradient Boosting suggest that nonlinear decision boundaries provide a more suitable representation of the neurophysiological patterns associated with ADHD than the linear classifier. Nevertheless, the partial overlap among the ROC curves also indicates that the performance differences between these classifiers should be interpreted together with the statistical analyses presented later in this section rather than solely on the basis of their AUC values.

### 4.4. Comparative Performance Analysis Across Classification Models

**Figure 9:**
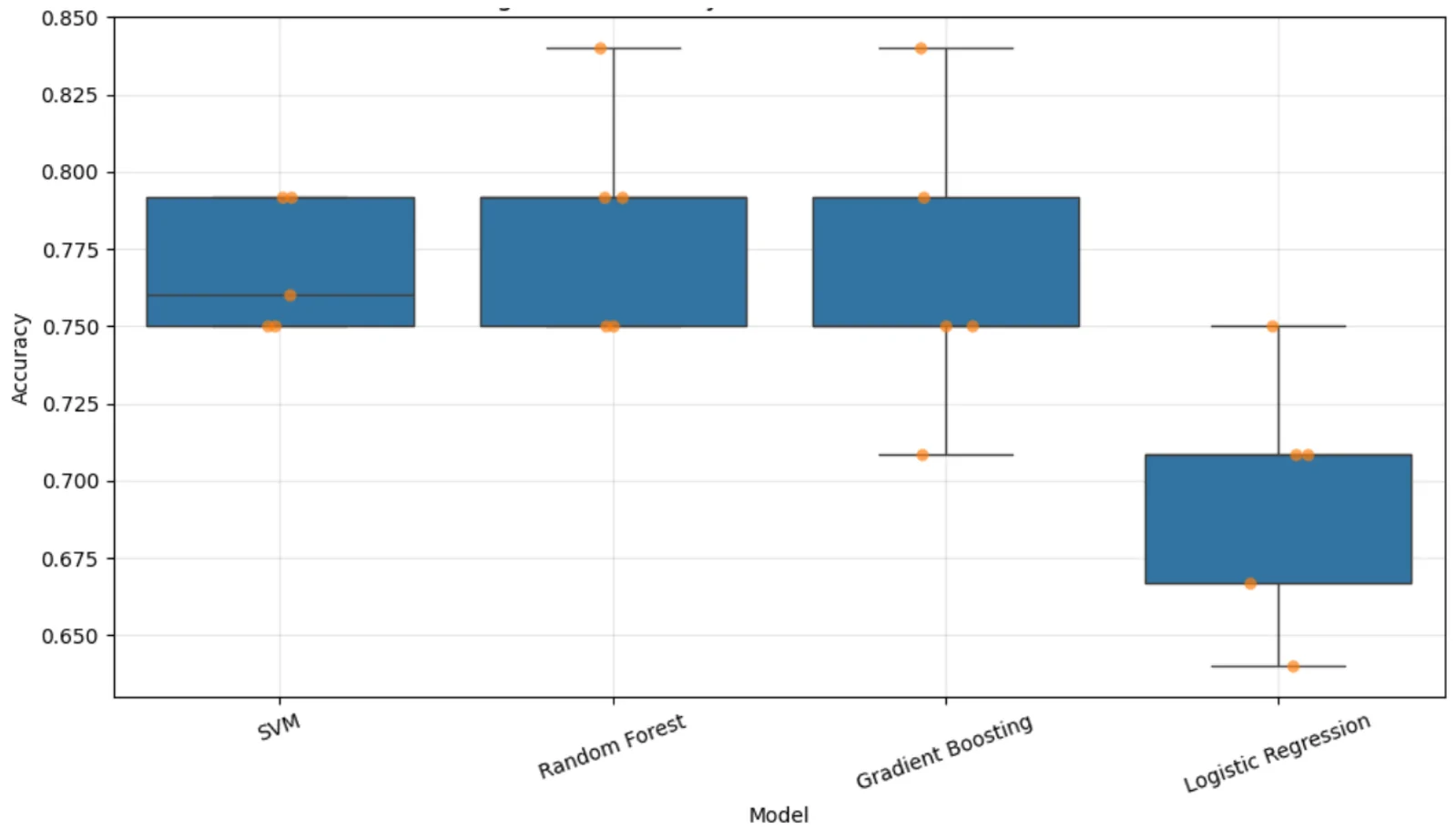
Distribution of classification accuracy across machine learning models using Stratified Group K-Fold cross-validation. Boxplots represent the variability of accuracy obtained for SVM, Random Forest, Gradient Boosting, and Logistic Regression across the evaluation folds.

Figure 9 illustrates the distribution of classification accuracy obtained for the four evaluated machine learning models using Stratified Group K-Fold cross-validation with strict subject-wise separation. Each boxplot summarizes the distribution of accuracy values across the validation folds, whereas the individual points correspond to the results obtained in each fold. This representation provides a visual assessment of both the central tendency and the variability of the evaluated classifiers.

The results indicate that Random Forest, SVM, and Gradient Boosting achieved comparable classification performance, exhibiting median accuracy values between approximately 0.77 and 0.81 together with relatively compact interquartile ranges, suggesting consistent performance across the validation folds. Among these classifiers, Random Forest showed the highest median accuracy, whereas SVM and Gradient Boosting demonstrated similar distributions with only moderate variability. Logistic Regression exhibited the lowest median accuracy together with a wider distribution of values, indicating reduced robustness when modeling the nonlinear characteristics of the EEG feature space.

Although the visual inspection of the boxplots suggests performance differences among the evaluated models, these differences cannot be inferred solely from their graphical distributions. Therefore, complementary statistical analyses based on Bootstrap confidence intervals, the Friedman test, and Wilcoxon post-hoc comparisons were subsequently performed to determine whether the observed variations were statistically significant. The corresponding results are presented in Table 3 and Table 4.

Overall, the comparative analysis indicates that nonlinear machine learning approaches, particularly Random Forest, SVM, and Gradient Boosting, consistently outperformed the linear Logistic Regression model for the classification of children with ADHD using EEG-derived features. However, as demonstrated by the subsequent statistical analysis, the observed performance differences among the three best-performing classifiers were relatively small, whereas Logistic Regression showed a statistically inferior performance compared with Random Forest and SVM.

### 4.5. Spatial EEG Channel Importance Analysis

**Figure 10:**
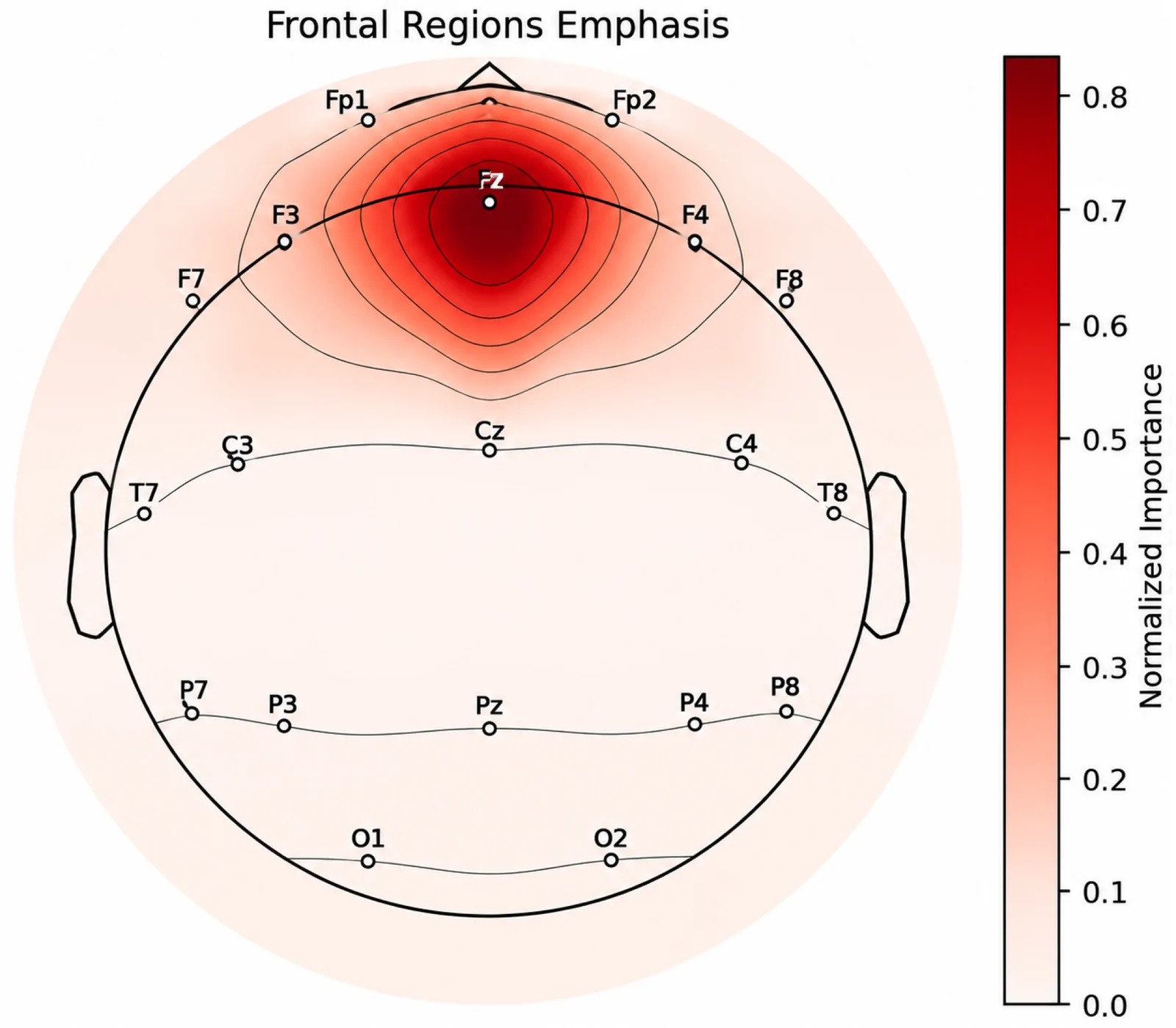
Topographic representation of the normalized EEG channel importance obtained from the Random Forest classifier, illustrating the relative contribution of each electrode to the ADHD versus Control classification task.

Figure 10 presents the topographic distribution of the relative importance assigned to each EEG channel by the Random Forest classifier based on the extracted feature set. The importance values represent the relative contribution of each channel to the classification process and do not correspond to measures of cortical activation or electrophysiological activity. Instead, they quantify the influence of the features extracted from each electrode on the final classification model.

The topographic representation reveals that the highest channel importance values are concentrated over the fronto-central scalp region, with electrode Fz showing the greatest contribution, followed by neighboring frontal electrodes including F3, Fp1, Fp2, and F4. This spatial distribution indicates that features derived from frontal EEG channels provided the most informative descriptors for discriminating between ADHD and control subjects within the proposed machine learning framework.

The predominance of frontal electrodes is consistent with previous neurophysiological studies reporting the involvement of fronto-central cortical regions in executive function, attentional regulation, inhibitory control, and working memory. Nevertheless, the present results should be interpreted as a model-based feature importance analysis rather than direct evidence of altered neural activity. Consequently, the observed spatial pattern reflects the relative contribution of the extracted EEG features to the classifier rather than the physiological intensity of cortical activation.

### 4.6. Quantitative Analysis of Frontal Channel Importance

**Figure 11:**
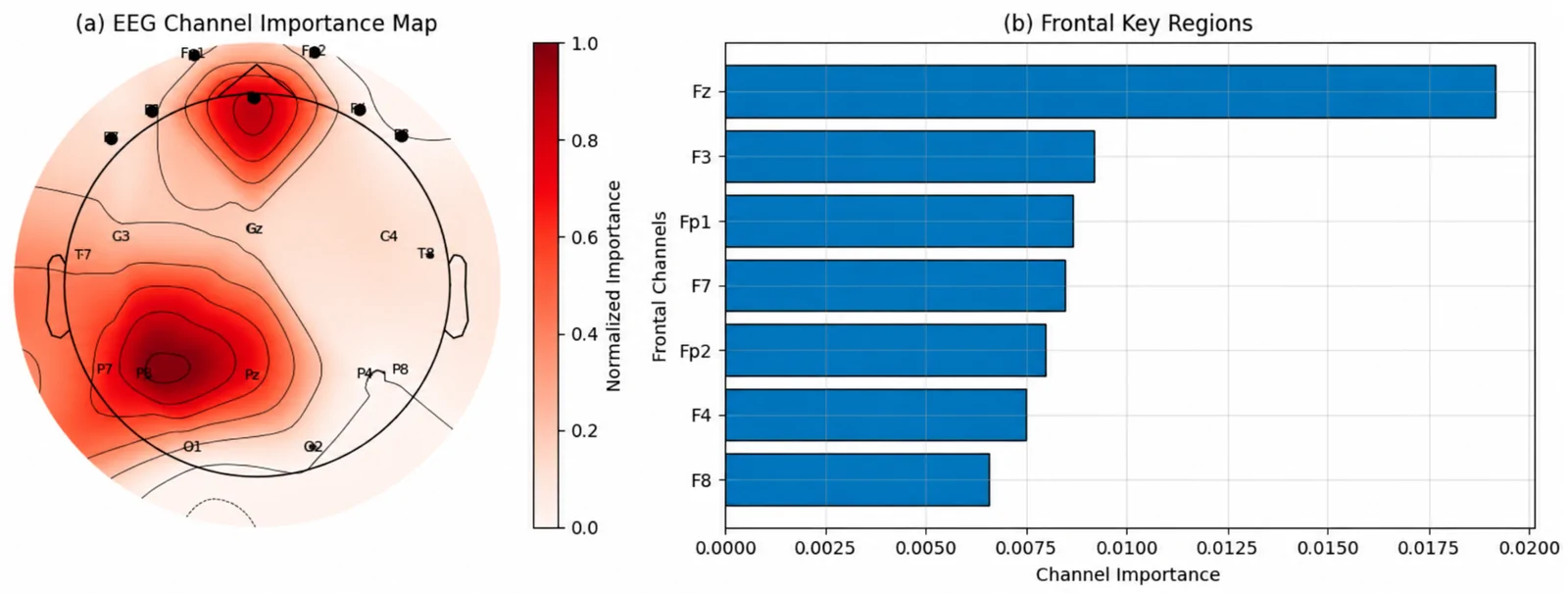
Relative importance ranking of frontal EEG channels showing the contribution of key frontal regions to ADHD classification performance.

Figure 11 presents the quantitative ranking of the frontal EEG channels according to their relative importance estimated by the Random Forest classifier. The graph compares the contribution of electrodes Fz, F3, Fp1, F7, Fp2, F4, and F8, providing a detailed view of the frontal channels that contributed most to the discrimination between ADHD and control subjects.

The results indicate that electrode Fz exhibited the highest relative importance among the analyzed frontal channels, followed by F3, Fp1, and F7, whereas Fp2, F4, and F8 showed comparatively lower importance values. This ranking reflects the relative contribution of the extracted features associated with each frontal electrode to the classification process rather than differences in electrophysiological activity or cortical activation.

The predominance of frontal electrodes is consistent with previous EEG studies reporting that executive function, attentional regulation, inhibitory control, and working memory are primarily associated with fronto-central cortical networks in children with ADHD. Consequently, the greater contribution of Fz and neighboring frontal channels suggests that features extracted from these regions contain more discriminative information for the proposed machine learning framework. However, these results should not be interpreted as direct evidence of altered neural activity but rather as an indication of the relative importance assigned by the classifier to the extracted EEG features.

Overall, the quantitative ranking of frontal electrodes complements the topographic representation shown in Figure 10 and provides additional evidence that frontal EEG-derived features constitute the most informative subset within the evaluated feature space for distinguishing ADHD from healthy control subjects under the adopted classification framework.

### 4.7. Feature Space Visualization Using t-SNE and UMAP

**Figure 12:**
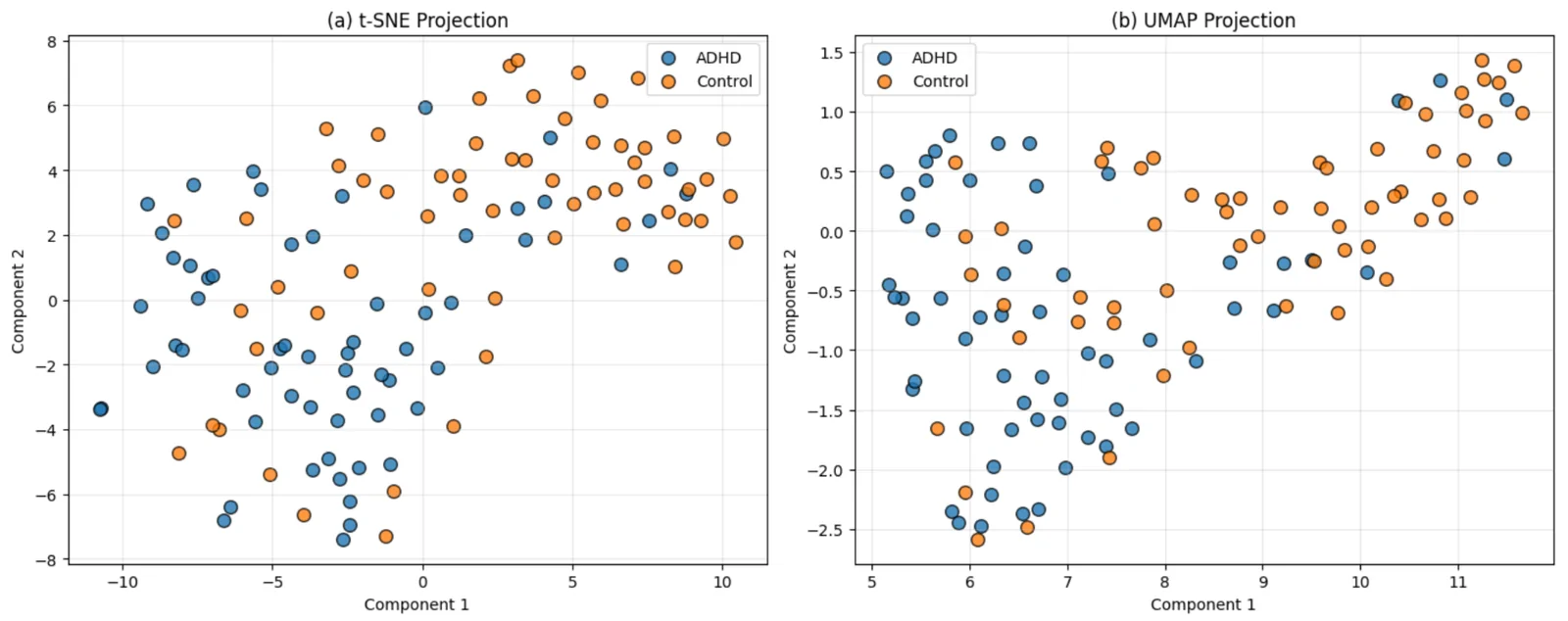
Two-dimensional visualization of the EEG feature space using (**a**) t-SNE and (**b**) UMAP. Both projections provide an exploratory representation of the distribution of ADHD and Control subjects based on the extracted EEG features.

Figure 12 presents the two-dimensional projection of the EEG feature space using two nonlinear dimensionality reduction techniques: t-SNE (t-distributed Stochastic Neighbor Embedding) and UMAP (Uniform Manifold Approximation and Projection). Both representations were used to visually explore the distribution of ADHD and Control subjects based on the extracted EEG features. These visualization techniques were employed exclusively for exploratory analysis and do not constitute quantitative measures of classification performance.

Figure 12a shows the projection obtained using t-SNE. A tendency toward differentiated clustering can be observed, with control subjects appearing more concentrated in the upper-right region of the embedded space, whereas ADHD subjects exhibit a more dispersed distribution across the central and lower regions. Although partial separation between both classes is evident, a region of overlap remains, reflecting the intrinsic variability of EEG signals and the heterogeneity of the pediatric population.

Figure 12b presents the projection generated using UMAP. Compared with t-SNE, UMAP produces a more structured organization of the feature space, showing a gradual tendency toward separation between the two diagnostic groups. Nevertheless, some overlap between ADHD and control subjects is still observed, indicating that the extracted features capture discriminative information while preserving the expected biological variability of EEG recordings.

Overall, both visualization techniques indicate that the extracted EEG features contain relevant information for differentiating ADHD and control subjects. However, these projections should be interpreted only as qualitative visualizations of the feature space rather than direct evidence of class separability. The quantitative evaluation of classifier performance is provided by the classification metrics, ROC analysis, and statistical tests presented in the following sections.

**Figure 13:**
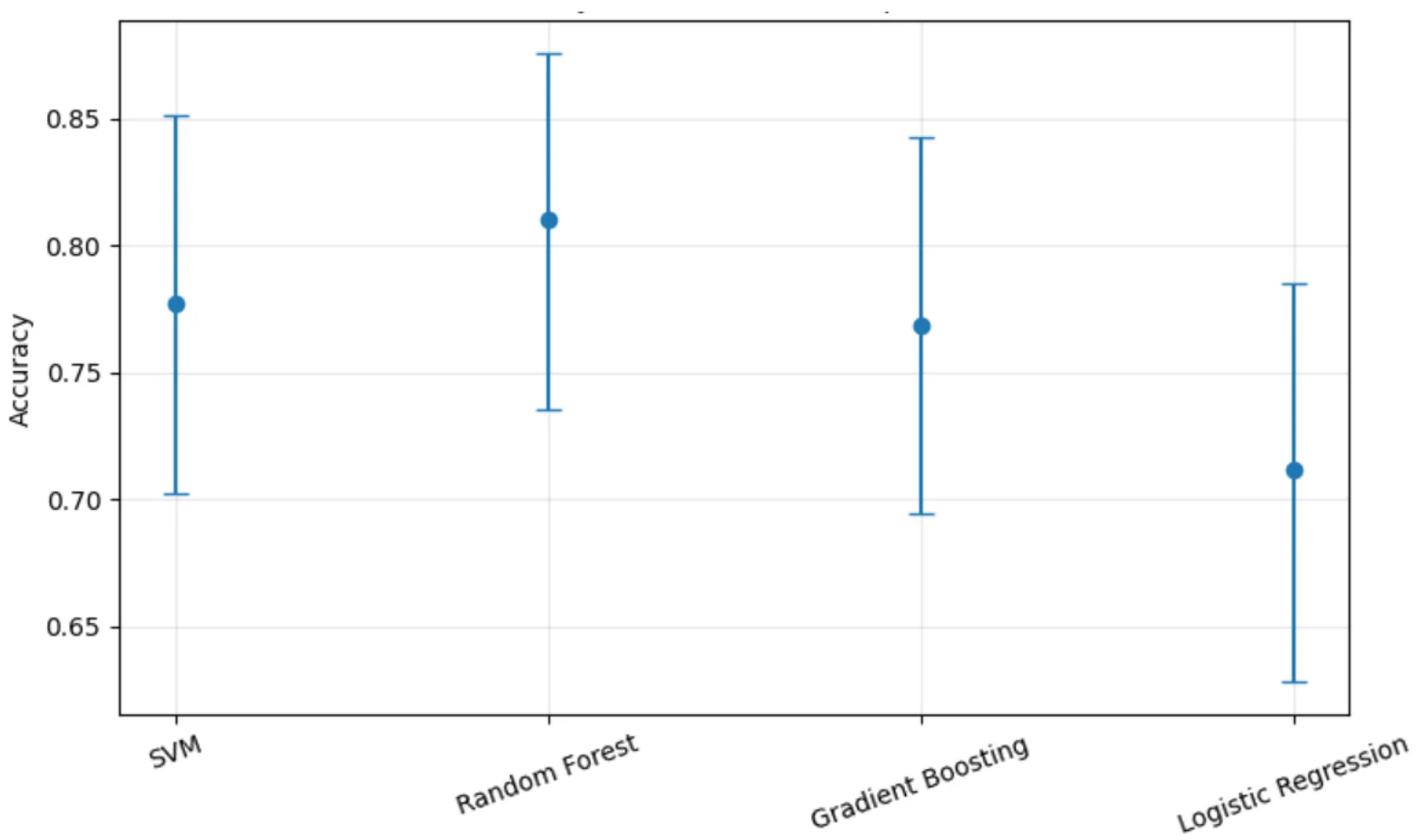
Mean classification accuracy together with the corresponding 95% bootstrap confidence intervals for the evaluated machine learning models.

Figure 13 presents the mean classification accuracy of the evaluated machine learning models together with their corresponding 95% bootstrap confidence intervals. Random Forest achieved the highest mean accuracy, followed by SVM and Gradient Boosting, whereas Logistic Regression exhibited the lowest overall performance. The bootstrap confidence intervals provide an estimate of the variability associated with the classification performance obtained through repeated resampling.

Random Forest and SVM exhibited relatively narrow confidence intervals with partial overlap, indicating consistent performance across bootstrap iterations. Gradient Boosting showed a similar trend, whereas Logistic Regression presented both lower mean accuracy and wider confidence intervals, suggesting greater variability in its predictive performance. These findings are consistent with the statistical comparisons presented in Table 4, where Logistic Regression was identified as significantly inferior to both Random Forest and SVM.

### 4.8. Comparative Performance of Classification Models

Table 2 summarizes the performance achieved by the four evaluated machine learning classifiers using Stratified Group K-Fold cross-validation with strict subject-wise separation. The reported metrics include Accuracy, Precision, Sensitivity, Specificity, F1-score, Balanced Accuracy, MCC, and ROC-AUC, providing a comprehensive assessment of the predictive performance of each model.

The comparative results indicate that Random Forest achieved the highest overall classification performance, obtaining the best values for Accuracy (0.8099), F1-score (0.8160), Balanced Accuracy (0.8097), and MCC (0.6204). These results suggest that the ensemble learning strategy provided an appropriate balance between correctly identifying children with ADHD and healthy control subjects. In contrast, the SVM classifier achieved the highest ROC-AUC (0.8527) together with the highest Sensitivity (0.8525), indicating strong discrimination capability across different decision thresholds. Gradient Boosting also demonstrated competitive performance across the evaluated metrics, whereas Logistic Regression exhibited the lowest overall classification performance.

Although Random Forest obtained the highest overall Accuracy, the numerical differences observed among Random Forest, SVM, and Gradient Boosting were relatively small. Therefore, these differences should not be interpreted solely on the basis of their point estimates. To evaluate the robustness of the obtained performance and determine whether the observed differences were statistically significant, additional analyses based on Bootstrap confidence intervals and non-parametric hypothesis testing were subsequently performed. The corresponding results are presented in Table 3 and Table 4.

Overall, the comparative evaluation indicates that nonlinear machine learning approaches consistently outperformed the linear Logistic Regression classifier for ADHD versus Control discrimination. These findings suggest that nonlinear decision boundaries provide a more suitable representation of the complex neurophysiological patterns extracted from multichannel EEG signals.

Table 3 summarizes the 95% bootstrap confidence intervals estimated for the principal performance metrics of the evaluated machine learning models. Unlike the point estimates reported in Table 2, the bootstrap analysis provides an estimation of the variability and reliability of the obtained performance through repeated resampling, allowing a more robust assessment of the stability of each classifier.

Random Forest achieved the highest mean Accuracy, F1-score, and Balanced Accuracy, while maintaining relatively narrow confidence intervals, indicating consistent classification performance across bootstrap resampling iterations. Similarly, SVM achieved the highest ROC-AUC, with confidence intervals comparable to those obtained by Random Forest, suggesting a similar level of robustness in terms of discriminative capability. Gradient Boosting also exhibited stable confidence intervals, whereas Logistic Regression presented lower mean performance together with comparatively wider confidence intervals.

An important observation is that the confidence intervals corresponding to Random Forest, SVM, and Gradient Boosting partially overlap for most evaluation metrics. This finding suggests that, although Random Forest achieved the highest numerical performance, the observed differences among these three classifiers may not necessarily represent statistically significant improvements. Consequently, formal hypothesis testing was performed to determine whether the apparent performance differences reflected true statistical differences rather than sampling variability.

Overall, the bootstrap analysis complements the point estimates reported in Table 2 by demonstrating that the three best-performing classifiers maintained relatively consistent performance across repeated resampling iterations, whereas Logistic Regression exhibited both lower predictive performance and greater variability.

Table 4 summarizes the results of the non-parametric statistical analysis performed to compare the classification performance of the evaluated machine learning models. Since all classifiers were evaluated using the same subject-wise cross-validation folds, the Friedman test was employed to determine whether the observed differences in classification performance were statistically significant across the evaluated models.

The Friedman test revealed statistically significant differences among the evaluated classifiers ($\chi^{2}=13.00$, p=0.0046), indicating that at least one classifier exhibited significantly different performance under the adopted validation protocol.

To identify the specific pairs of classifiers responsible for these differences, post-hoc pairwise comparisons were subsequently performed using the Wilcoxon signed-rank test. The results showed statistically significant differences between Random Forest and Logistic Regression (p=0.0047), as well as between SVM and Logistic Regression (p=0.0114). In contrast, no statistically significant differences were observed among Random Forest, SVM, and Gradient Boosting (p>0.05), indicating that these three classifiers achieved statistically comparable performance despite the small numerical differences observed in their evaluation metrics.

The statistical analysis complements the Bootstrap confidence interval estimation by showing that the numerically superior performance achieved by Random Forest should be interpreted together with its associated uncertainty and statistical significance. Consequently, although Random Forest obtained the highest overall Accuracy, the absence of statistically significant differences with respect to SVM and Gradient Boosting suggests that these three nonlinear classifiers provide comparable predictive performance for ADHD versus Control discrimination using the analyzed EEG features. Conversely, Logistic Regression consistently exhibited significantly lower performance, highlighting the limitations of linear decision boundaries for modeling the complex neurophysiological patterns present in pediatric EEG signals.

Overall, the combination of performance metrics, Bootstrap confidence intervals, and non-parametric statistical testing provides a comprehensive evaluation of classifier performance, indicating that Random Forest, SVM, and Gradient Boosting showed comparable performance for EEG-based ADHD classification, whereas Logistic Regression exhibited lower predictive performance under the adopted experimental conditions.

### 4.9. Individualized Frequency-Band Analysis

To evaluate the effect of inter-individual variability in EEG spectral characteristics, an additional analysis was performed using frequency bands defined relative to each participant’s IAF. The mean IAF was 8.8320 Hz for the ADHD group and 8.8833 Hz for the Control group, indicating highly similar average dominant alpha frequencies between the two groups in the analyzed dataset. Table 5 compares the classification performance obtained using the conventional fixed frequency bands with that obtained using the individualized IAF-based frequency bands. Under the individualized analysis, SVM achieved the highest accuracy (0.7438), followed by Random Forest (0.7273), Gradient Boosting (0.7025), and Logistic Regression (0.6942). SVM also achieved the highest ROC-AUC (0.7888), closely followed by Random Forest (0.7880). Compared with the conventional fixed-band analysis, individualized frequency bands did not improve classification performance. Accuracy decreased by 0.0331 for SVM, 0.0826 for Random Forest, 0.0661 for Gradient Boosting, and 0.0165 for Logistic Regression. These results indicate that accounting for subject-specific alpha frequency modifies the extracted spectral representation but does not provide an improvement in ADHD versus Control classification performance in the present dataset.

### 4.10. Computational Cost Analysis

To complement the predictive performance analysis, the computational cost of the four evaluated classifiers was quantified under the Leave-One-Subject-Out (LOSO) validation protocol. For each LOSO iteration, the training time and the inference time required to classify the held-out subject were measured using the same preprocessing, feature extraction, and model configuration adopted in the main experiments. The reported values correspond to the mean and standard deviation obtained across the 121 LOSO iterations.

Table 6 summarizes the computational performance of the evaluated models. Gradient Boosting exhibited the highest mean training time (31.5860 s), followed by SVM (23.1698 s) and Random Forest (20.3046 s), whereas Logistic Regression required substantially less training time (0.2738 s). In terms of subject-level inference, Gradient Boosting achieved the lowest mean prediction time (2.6547 ms/subject), followed by Logistic Regression (4.0498 ms/subject) and SVM (97.0299 ms/subject). Random Forest showed the highest mean inference time, reaching 162.4704 ms per subject.

These results indicate that the classifiers exhibit different trade-offs between predictive performance and computational efficiency. Although Random Forest achieved the highest overall classification accuracy, its subject-level inference time was the largest among the evaluated models. In contrast, Gradient Boosting provided substantially faster inference while maintaining competitive classification performance, whereas Logistic Regression showed the lowest training cost but also the lowest predictive performance. Importantly, all evaluated models produced subject-level predictions within fractions of a second, suggesting that the computational requirements of the classification stage are compatible with offline EEG analysis and potentially with decision-support applications after feature extraction.

## 5. Discussion

The results obtained in this study indicate that EEG-derived features contain relevant information for distinguishing children with ADHD from healthy control subjects using machine learning techniques. Overall, the evaluated models achieved consistent classification performance, with ROC-AUC values ranging from 0.7907 to 0.8527 and classification accuracies between 0.7107 and 0.8099. These results support the initial hypothesis that quantitative features extracted from multichannel EEG signals can contribute to the discrimination between ADHD and control subjects. However, the observed classification performance should not be interpreted as direct evidence of specific neurophysiological alterations or as a diagnostic biomarker, since the present study evaluates the discriminative contribution of EEG-derived features within a machine learning framework.

One of the main findings concerns the performance of nonlinear classification models. The SVM classifier achieved the highest ROC-AUC value (0.8527), whereas Random Forest obtained the highest overall accuracy (0.8099), F1-score (0.8160), and MCC (0.6204). This behavior suggests that nonlinear classifiers may provide a more suitable representation of the relationships contained within the extracted EEG feature space than the linear Logistic Regression model. Nevertheless, the numerical superiority of Random Forest, SVM, and Gradient Boosting should be interpreted cautiously. The Friedman test revealed an overall statistically significant difference among classifiers ($\chi^{2}=13.00$, p=0.0046); however, the Wilcoxon post-hoc comparisons showed no statistically significant differences among Random Forest, SVM, and Gradient Boosting (p>0.05). Significant differences were observed only between Random Forest and Logistic Regression (p=0.0047) and between SVM and Logistic Regression (p=0.0114). Therefore, the three nonlinear classifiers can be considered statistically comparable under the adopted evaluation protocol, despite their numerical differences in individual performance metrics.

These findings are consistent with the general tendency reported in previous EEG-based ADHD classification studies, in which nonlinear and ensemble-based approaches have shown competitive performance for modeling complex EEG-derived feature spaces. The bootstrap analysis further supports a cautious interpretation of the differences among the best-performing classifiers, as their 95% confidence intervals showed substantial overlap. The classification performance obtained in the present study also is comparable to values reported in previous EEG-based ADHD classification research, although direct comparisons should be made cautiously because performance estimates depend strongly on the dataset, participant characteristics, feature extraction procedures, and validation strategy. In addition to predictive performance, the channel-importance analysis identified a greater model-derived contribution from frontal and fronto-central electrodes, particularly Fz, F3, Fp1, F7, Fp2, F4, and F8. Importantly, these importance values represent the relative contribution of EEG-derived features to the classifier and should not be interpreted as measurements of cortical activation or as direct evidence of regional neurophysiological abnormalities.

The predominance of Fz in the channel-importance analysis is noteworthy because frontal and fronto-central EEG regions have been widely associated with cognitive processes involving attentional control, inhibitory processing, and executive regulation. In the present study, however, the greater importance assigned to Fz should be interpreted specifically as a model-derived measure indicating that features extracted from this electrode contributed more strongly to the classification decision. It does not represent increased cortical activation, nor does it provide direct evidence of an electrophysiological abnormality localized to this region.

The higher relative contribution of Fz and neighboring frontal electrodes is nevertheless consistent with previous EEG studies reporting the relevance of fronto-central activity in the neurophysiological characterization of ADHD. Therefore, the present findings suggest that EEG-derived features from frontal and fronto-central channels may contain useful discriminative information for distinguishing ADHD from control subjects within the proposed machine learning framework. Further studies using independent cohorts and dedicated neurophysiological analyses are required before these channel-specific patterns can be considered reliable biomarkers of ADHD.

The two-dimensional representations obtained using t-SNE and UMAP provide an exploratory visualization of the structure of the extracted EEG feature space. Both projections showed a partial tendency toward separation between ADHD and Control subjects, although a considerable degree of overlap between the two groups remained evident. These visualizations should be interpreted as qualitative and exploratory representations rather than as statistical evidence of class separability, since nonlinear dimensionality-reduction techniques such as t-SNE and UMAP can emphasize local or global structures depending on their parameterization. Therefore, the observed clustering patterns are used only as complementary visual support for understanding the organization of the extracted feature space and not as independent evidence of neurophysiological differences between ADHD and Control subjects.

More importantly, the predictive performance of the classifiers was assessed using a subject-wise validation strategy in which EEG windows belonging to the same participant were kept within the same partition, thereby preventing information leakage between training and testing data. This methodological constraint is particularly relevant for EEG classification because multiple highly correlated windows can be generated from a single recording. Consequently, subject-level separation provides a more conservative estimate of generalization to previously unseen participants than random window-level splitting. Nevertheless, the reported results represent internal validation on a single dataset and should not be interpreted as direct evidence of performance in real-world clinical settings; external validation on independent cohorts remains necessary to establish the generalizability of the proposed approach.

From a clinical perspective, the findings suggest the potential of EEG-derived features as a complementary source of quantitative information for the neurophysiological characterization of ADHD. However, the present results should not be interpreted as demonstrating the clinical diagnostic utility of the proposed models. Clinical diagnosis of ADHD requires comprehensive behavioral and neuropsychological assessment, and the classification framework evaluated in this study has not yet been externally validated in independent clinical populations. Therefore, its current role should be considered exploratory and methodological rather than diagnostic.

The model-derived contribution of frontal and fronto-central EEG channels may provide complementary information for investigating patterns associated with attentional and executive processes. Nevertheless, these channel-importance patterns represent discriminative contributions within the machine learning model and should not be considered validated neurophysiological biomarkers. Further validation using independent cohorts and complementary neurophysiological analyses is required before their potential clinical relevance can be established.

Despite the encouraging results, several limitations should be considered. First, the analysis was performed using relatively simple statistical and spectral features, including the mean, standard deviation, and theta-, alpha-, and beta-band power. Although these features provided useful discriminative information, they do not fully characterize the complex spatial, temporal, and nonlinear dynamics of EEG activity. Future studies could therefore investigate complementary representations based on functional connectivity, brain network analysis, entropy measures, nonlinear dynamical descriptors, and advanced time–frequency features.

Second, the proposed framework was evaluated on a single dataset comprising 121 participants (61 ADHD and 60 Control subjects). Although strict subject-wise separation was adopted to prevent information leakage between training and testing data, this procedure represents internal validation and does not replace evaluation on an independent cohort. Future investigations should validate the proposed models on independent and multicenter datasets to assess their robustness against differences in participant demographics, clinical characteristics, EEG acquisition systems, recording protocols, and experimental conditions.

The bootstrap analysis provided additional information regarding the uncertainty associated with the classification performance of the evaluated models. Random Forest achieved a mean Accuracy of 0.8100 (95% CI: 0.7355–0.8760), while SVM achieved the highest ROC-AUC of 0.8528 (95% CI: 0.7776–0.9161). However, the confidence intervals of Random Forest, SVM, and Gradient Boosting showed substantial overlap across the principal evaluation metrics, indicating that the numerical differences among these classifiers should be interpreted cautiously. The additional subject-specific spectral analysis provides further insight into the influence of inter-individual EEG frequency variability on classification performance. The mean individual alpha frequency (IAF) was 8.8320 Hz in the ADHD group and 8.8833 Hz in the Control group, showing only a small difference in the average dominant alpha frequency between groups in the present dataset. When spectral features were recalculated using individualized IAF-based theta, alpha, and beta bands, classification performance decreased relative to the conventional fixed-band analysis. SVM achieved the highest accuracy under the individualized approach (0.7438), compared with 0.7769 using fixed bands, whereas Random Forest decreased from 0.8099 to 0.7273. Similar reductions were observed for Gradient Boosting and Logistic Regression. These findings indicate that accounting for subject-specific alpha frequency altered the spectral representation but did not improve classification performance in this dataset. Importantly, this result does not exclude the neurophysiological relevance of individualized frequency boundaries; rather, it suggests that their contribution to ADHD discrimination may depend on the recording paradigm, spectral estimation procedure, participant characteristics, and feature representation employed. This interpretation was further supported by the statistical comparison. The Friedman test revealed a statistically significant overall difference among the evaluated classifiers ($\chi^{2}=13.0000$, p=0.0046). Nevertheless, the post-hoc Wilcoxon signed-rank tests showed no statistically significant differences between Random Forest and SVM (p=0.2482), Gradient Boosting and Random Forest (p=0.0588), or Gradient Boosting and SVM (p=0.7389). Significant differences were observed only between Random Forest and Logistic Regression (p=0.0047) and between SVM and Logistic Regression (p=0.0114), whereas the difference between Gradient Boosting and Logistic Regression did not reach statistical significance (p=0.0522).

Taken together, these findings indicate that Random Forest, SVM, and Gradient Boosting exhibited statistically comparable performance under the adopted evaluation protocol, despite the numerical differences observed in individual metrics. Therefore, Random Forest should not be interpreted as statistically superior to SVM or Gradient Boosting solely because it achieved the highest Accuracy. In contrast, Logistic Regression generally showed the lowest predictive performance, with statistically significant differences relative to Random Forest and SVM.

As a future research direction, it would be particularly valuable to incorporate deep learning architectures specifically designed for spatiotemporal EEG modeling, such as EEGNet, CNN–LSTM networks, Transformers, or hybrid attention-based architectures. These approaches could enable the automatic learning of spatial, spectral, and temporal EEG representations while reducing the dependence on manually defined features. However, their evaluation should preserve strict subject-wise separation and include external validation to determine whether the potential improvements generalize to independent populations.

Furthermore, future studies should investigate complementary neurophysiological representations, including functional connectivity, entropy-based measures, brain network properties, and nonlinear EEG descriptors. These analyses could provide additional information regarding the relationships between discriminative EEG patterns and the neural processes associated with attention and executive function in ADHD.

The incorporation of explainable artificial intelligence approaches, such as SHAP-based feature attribution, could also improve the transparency of classification models by identifying the features and EEG channels that most strongly influence individual predictions. Such analyses would complement the channel-importance approach used in the present study and could provide a more detailed assessment of model behavior.

Finally, combining EEG-derived information with neuropsychological, behavioral, and other neurobiological variables may contribute to the development of more comprehensive multimodal frameworks. Nevertheless, prospective studies involving larger and independent clinical cohorts will be required before such approaches can be considered for clinical decision-support applications. Therefore, future work should prioritize external validation, reproducibility across acquisition settings, and assessment of model robustness rather than interpreting classification performance alone as evidence of diagnostic utility.

### Comparison with Previous Studies

Table 7 compares the present results with previous studies that employed conventional machine learning techniques for EEG-based ADHD classification. The reviewed approaches include SVM, XGBoost, Naive Bayes, and feature-selection methods applied to statistical, morphological, spectral, and quantitative EEG representations. Considerable methodological variability exists among these studies, particularly in preprocessing, feature extraction, dimensionality reduction, segmentation, and validation procedures.

Several previous studies reported higher classification accuracies than the 80.99% obtained with Random Forest in the present study. However, direct numerical comparison should be interpreted with caution because the studies differ substantially in preprocessing, feature extraction, segmentation, sample composition, and validation strategy. In particular, performance estimates obtained from segment- or epoch-based evaluation are not directly comparable with protocols that enforce strict separation of participants between training and testing sets.

A relevant methodological aspect of the present study is the use of Stratified Group K-Fold cross-validation with strict subject-wise separation. EEG windows belonging to the same participant were never simultaneously included in the training and testing partitions, reducing the risk of information leakage associated with correlated segments from the same subject. This distinction is particularly relevant when comparing studies using the same public ADHD EEG dataset, since differences in validation strategy can substantially affect the reported classification performance.

Therefore, the objective of the present study was not to outperform previously reported classification accuracies, but to evaluate the discriminative capability of a relatively simple and interpretable set of statistical and spectral EEG features under a strict subject-wise evaluation framework. The obtained 80.99% accuracy should consequently be interpreted within the context of the adopted validation protocol rather than as a direct performance ranking against previous studies.

In addition to classification performance, the proposed framework incorporated statistical uncertainty analysis and model-based channel interpretation. The channel-importance analysis identified greater contributions from frontal and fronto-central electrodes, particularly Fz. These results represent classifier-specific feature contributions and should not be interpreted as direct measurements of cortical activation or validated neurophysiological biomarkers. External validation using independent and multicenter EEG datasets remains necessary to determine the reproducibility and generalizability of the proposed approach.

## 6. Conclusions

This study investigated the discrimination of children with ADHD and healthy control subjects using multichannel EEG-derived features and conventional machine learning classifiers. The results indicate that the extracted statistical and spectral features contain relevant discriminative information for this classification task. Among the evaluated models, Random Forest achieved the highest overall Accuracy (0.8099), F1-score (0.8160), Balanced Accuracy (0.8097), and MCC (0.6204), whereas SVM achieved the highest Sensitivity (0.8525) and ROC-AUC (0.8527). Gradient Boosting also showed competitive performance, while Logistic Regression obtained the lowest overall classification results.

Importantly, the numerical differences among the best-performing classifiers should be interpreted together with their associated statistical uncertainty. The 95% bootstrap confidence intervals showed substantial overlap among Random Forest, SVM, and Gradient Boosting. Although the Friedman test identified an overall statistically significant difference among the evaluated classifiers ($\chi^{2}=13.0000$, p=0.0046), the Wilcoxon post-hoc comparisons revealed no statistically significant differences among Random Forest, SVM, and Gradient Boosting (p>0.05). Significant differences were observed between Random Forest and Logistic Regression (p=0.0047) and between SVM and Logistic Regression (p=0.0114). Therefore, the three nonlinear classifiers exhibited statistically comparable performance under the adopted evaluation conditions, despite differences in their individual performance metrics.

The channel-importance analysis identified a greater relative contribution from frontal and fronto-central electrodes, particularly Fz, followed by F3, Fp1, F7, Fp2, F4, and F8. These results indicate that features extracted from these channels contributed more strongly to the classification decisions of the model. However, channel importance should not be interpreted as a direct measurement of cortical activation or as evidence of a validated neurophysiological biomarker. The observed frontal and fronto-central distribution is consistent with brain regions frequently associated in the literature with attentional and executive processes, but independent neurophysiological analyses and external cohorts are required to confirm the biological relevance and reproducibility of these patterns.

The temporal and spectral representations provided complementary descriptions of the EEG signals, while the functional connectivity representation illustrated inter-channel relationships within the analyzed recordings. Similarly, t-SNE and UMAP showed partial organization of ADHD and Control samples within the reduced feature space. These dimensionality-reduction results should be considered exploratory visualizations rather than statistical evidence of class separability or direct evidence of neurophysiological differences between groups.

A relevant methodological aspect of the proposed framework was the preservation of strict subject-wise separation during model evaluation, preventing EEG windows belonging to the same participant from being simultaneously included in training and testing partitions. This strategy reduces the risk of information leakage associated with highly correlated EEG segments and provides a more rigorous assessment of classification performance on unseen subjects. Nevertheless, the present results constitute internal validation on a single dataset of 121 participants (61 ADHD and 60 Control subjects) and therefore should not be interpreted as evidence of generalization to independent clinical populations.

An additional subject-specific spectral analysis was performed to account for inter-individual variability in EEG frequency boundaries. The mean individual alpha frequency (IAF) was 8.8320 Hz for the ADHD group and 8.8833 Hz for the Control group. When the spectral features were recalculated using individualized IAF-based theta, alpha, and beta bands, the classification performance did not improve relative to the conventional fixed-band analysis. Under the individualized approach, SVM achieved the highest Accuracy (0.7438), compared with 0.7769 using fixed bands, while Random Forest decreased from 0.8099 to 0.7273. These findings indicate that individualized frequency boundaries modified the spectral representation but did not provide an additional classification advantage in the present dataset.

The computational cost analysis further demonstrated important differences among the evaluated classifiers. Logistic Regression required the lowest mean training time (0.2738 s), whereas Gradient Boosting exhibited the lowest mean subject-level inference time (2.6547 ms/subject). Random Forest, despite achieving the highest overall classification accuracy, required a mean training time of 20.3046 s and the highest mean inference time (162.4704 ms/subject). SVM required 23.1698 s for training and 97.0299 ms/subject for inference, while Gradient Boosting presented the highest training cost (31.5860 s). Despite these differences, all models produced subject-level predictions within fractions of a second, indicating that the classification stage itself has relatively modest computational requirements for offline EEG analysis.

Overall, the findings support the feasibility of using EEG-derived features together with machine learning techniques for the computational discrimination between ADHD and Control subjects. However, the proposed framework should currently be regarded as a research-oriented classification approach rather than a clinically validated diagnostic system. The results do not establish EEG-derived features as diagnostic biomarkers, nor do they demonstrate clinical utility for ADHD diagnosis. External validation, reproducibility across acquisition settings, and prospective evaluation in independent clinical populations remain necessary before considering translation into clinical decision-support applications.

Future research should therefore prioritize validation using larger independent and multicenter datasets, together with the investigation of complementary EEG representations, including functional connectivity, entropy-based measures, nonlinear descriptors, and advanced time–frequency features. Deep learning architectures specifically designed for spatiotemporal EEG analysis, such as EEGNet, CNN–LSTM models, Transformers, and attention-based architectures, could also be investigated while preserving strict subject-wise evaluation. Finally, the integration of explainable artificial intelligence methods and multimodal information combining EEG with behavioral, neuropsychological, and other neurobiological variables may contribute to a more comprehensive characterization of ADHD. Such developments should be accompanied by rigorous external validation before their potential clinical applicability can be established.

## Figures and Tables

**Table 1:** Description of the EEG dataset and acquisition protocol used in this study.

Parameter	Description
Dataset composition	121 participants: 61 children with ADHD and 60 healthy controls
Age range	7–12 years
Participant sex	Boys and girls; the available dataset description does not provide the exact sex distribution for each group
ADHD diagnosis	ADHD participants were diagnosed by an experienced psychiatrist according to DSM-IV criteria
Medication	Children with ADHD had received Ritalin for up to 6 months
Control group criteria	Control participants had no history of psychiatric disorders, epilepsy, or reported high-risk behaviors
EEG system	International 10–20 electrode placement system
Number of EEG channels	19 channels
EEG channels	Fz, Cz, Pz, C3, T3, C4, T4, Fp1, Fp2, F3, F4, F7, F8, P3, P4, T5, T6, O1, O2
Reference electrodes	A1 and A2, located on the earlobes
Sampling frequency	128 Hz
Recording protocol	Visual attention task based on counting cartoon characters displayed on a screen
Visual stimulus	Each image contained a randomly selected number of cartoon characters ranging from 5 to 16
Task procedure	Participants were instructed to count the characters presented in each image. A new image was displayed immediately after each response to maintain continuous visual stimulation
Recording duration	Variable across participants and dependent on individual response speed during the visual attention task

**Table 2:** Performance metrics obtained by the evaluated classification models using Stratified Group K-Fold cross-validation.

Model	Accuracy	Precision	Sensitivity	Specificity	F1-Score	Balanced Accuracy	MCC	ROC-AUC
Random Forest	0.8099	0.7969	0.8361	0.7833	0.8160	0.8097	0.6204	0.8347
SVM	0.7769	0.7429	0.8525	0.7000	0.7939	0.7762	0.5594	0.8527
Gradient Boosting	0.7686	0.7538	0.8033	0.7333	0.7778	0.7683	0.5381	0.8224
Logistic Regression	0.7107	0.6970	0.7541	0.6667	0.7244	0.7104	0.4225	0.7907
**Average**	**0.7665**	**0.7476**	**0.8115**	**0.7208**	**0.7780**	**0.7662**	**0.5351**	**0.8251**

**Table 3:** Bootstrap 95% confidence intervals for the performance metrics of the evaluated machine learning models.

Model	Accuracy (95% CI)	F1-Score (95% CI)	Balanced Accuracy (95% CI)	ROC-AUC (95% CI)
Random Forest	**0.8100 [0.7355–0.8760]**	**0.8149 [0.7317–0.8855]**	**0.8098 [0.7366–0.8770]**	0.8348 [0.7545–0.9049]
SVM	0.7770 [0.7025–0.8512]	0.7928 [0.7119–0.8633]	0.7764 [0.6993–0.8489]	**0.8528 [0.7776–0.9161]**
Gradient Boosting	0.7687 [0.6942–0.8430]	0.7766 [0.6885–0.8507]	0.7684 [0.6900–0.8410]	0.8226 [0.7416–0.8947]
Logistic Regression	0.7115 [0.6281–0.7851]	0.7235 [0.6299–0.8032]	0.7111 [0.6275–0.7865]	0.7910 [0.7055–0.8647]

**Table 4:** Statistical comparison of the evaluated classification models using the Friedman test and Wilcoxon post-hoc pairwise comparisons.

Overall Statistical Test		
Test	Statistic	p-Value
Friedman	13.0000	**0.0046**
**Post-hoc Pairwise Comparisons**		
**Comparison**	**p-value**	**Result**
RF vs. LR	**0.0047**	Significant
SVM vs. LR	**0.0114**	Significant
RF vs. SVM	0.2482	Not significant
GB vs. RF	0.0588	Not significant
GB vs. LR	0.0522	Not significant
GB vs. SVM	0.7389	Not significant

**Table 5:** Comparison of classification performance using conventional fixed frequency bands and individualized IAF-based frequency bands.

Model	Fixed-Band Accuracy	IAF Accuracy	Δ Accuracy	Fixed-Band F1	IAF F1	IAF ROC-AUC
SVM	0.7769	0.7438	−0.0331	0.7939	0.7597	0.7888
Random Forest	0.8099	0.7273	−0.0826	0.8160	0.7556	0.7880
Gradient Boosting	0.7686	0.7025	−0.0661	0.7778	0.7313	0.7743
Logistic Regression	0.7107	0.6942	−0.0165	0.7244	0.7132	0.7757

**Table 6:** Computational cost of the evaluated machine learning models under Leave-One-Subject-Out validation. Values represent mean ± standard deviation across 121 subject-level iterations.

Model	Training Time (s)	Prediction Time (ms/Subject)
SVM	23.1698 ± 0.8382	97.0299 ± 44.4572
Random Forest	20.3046 ± 1.1120	162.4704 ± 14.7558
Gradient Boosting	31.5860 ± 0.7140	2.6547 ± 0.6410
Logistic Regression	0.2738 ± 0.1101	4.0498 ± 1.3520

**Table 7:** Comparison of EEG-based ADHD classification studies using machine learning techniques.

Study	Dataset/Subjects	Main Method	EEG Features	Validation Strategy	Accuracy (%)
Maniruzzaman et al. [54]	61 ADHD/60 Control	LASSO + SVM	Morphological and time-domain features	Cross-validation after feature selection	94.20
Ahire et al. [55]	61 ADHD/60 Control	Bernoulli Naive Bayes	Morphological and PSD features with PCA	Train–test classification with PCA-based feature reduction	96.00
García-Ponsoda et al. [56]	Pediatric ADHD/Control	SVM, KNN, XGBoost	Kurtosis, Katz FD, Delta, Theta, and Alpha power	Segment-based evaluation under different temporal segmentation configurations	86.10
Kim et al. [57]	107 ADHD/61 Neurotypical	XGBoost	Delta, Theta, Alpha, Beta, and Gamma qEEG features	Leave-One-Subject-Out (LOSO) cross-validation	90.81
**Proposed Study**	**61 ADHD/60 Control**	**SVM, RF, GB, LR**	**Mean, SD, Theta, Alpha, and Beta power**	**Stratified Group K-Fold with strict subject-wise separation**	**80.99**

## Data Availability

The database used in this study is publicly available and can be accessed through the following repository: EEG Dataset for ADHD: https://www.kaggle.com/datasets/danizo/eeg-dataset-for-adhd [53], accessed on 1 September 2026.
